# Transcriptome analysis of an *mvp* mutant reveals important changes in global gene expression and a role for methyl jasmonate in vernalization and flowering in wheat

**DOI:** 10.1093/jxb/eru102

**Published:** 2014-03-28

**Authors:** Amadou Oury Diallo, Zahra Agharbaoui, Mohamed A. Badawi, Mohamed Ali Ali-Benali, Amira Moheb, Mario Houde, Fathey Sarhan

**Affiliations:** Département des Sciences biologiques Université du Québec à Montréal, CP 8888, Succursale Centre-Ville Montréal, Québec H3C 3P8Canada

**Keywords:** Biotic stress, flowering, maintained vegetative phase, methyl jasmonate, vernalization, wheat.

## Abstract

The einkorn wheat mutant *mvp-1* (*maintained vegetative phase 1*) has a non-flowering phenotype caused by deletions including, but not limited to, the genes *CYS*, *PHYC*, and *VRN1*. However, the impact of these deletions on global gene expression is still unknown. Transcriptome analysis showed that these deletions caused the upregulation of several pathogenesis-related (PR) and jasmonate-responsive genes. These results suggest that jasmonates may be involved in flowering and vernalization in wheat. To test this hypothesis, jasmonic acid (JA) and methyl jasmonate (MeJA) content in *mvp* and wild-type plants was measured. The content of JA was comparable in all plants, whereas the content of MeJA was higher by more than 6-fold in *mvp* plants. The accumulation of MeJA was also observed in vernalization-sensitive hexaploid winter wheat during cold exposure. This accumulation declined rapidly once plants were deacclimated under floral-inductive growth conditions. This suggests that MeJA may have a role in floral transition. To confirm this result, we treated vernalization-insensitive spring wheat with MeJA. The treatment delayed flowering with significant downregulation of both *TaVRN1* and *TaFT1* genes. These data suggest a role for MeJA in modulating vernalization and flowering time in wheat.

## Summary

Molecular and physiological analyses of a wheat *mvp* mutant, and winter and spring wheats suggest that methyl jasmonate is involved in modulating vernalization and floral transition in wheat.

## Introduction

Flowering is one of the most crucial developmental programmes that plants use to ensure survival and reproductive success. These programmes are regulated by both environmental and internal developmental factors. In *Arabidopsis*, there are four floral promotion pathways: the vernalization, the photoperiod, the autonomous, and the gibberellin (GA) pathways (Boss *et al.*, 2004). Vernalization and photoperiod pathways are integrated by *FLOWERING LOCUS T* (*FT*) and its activity is directly suppressed by *FLOWERING LOCUS C* (*FLC*) (Searle *et al.*, 2006). FT acts as a long-distance flowering signal (*florigen*) and encodes a protein with similarity to the animal Raf kinase inhibitor-like protein (RKIP) (Kardailsky *et al.*, 1999; Kobayashi *et al.*, 1999). Expression of *CONSTANS* (*CO*) peaks during the light period under long-day (LD) conditions, resulting in early flowering by activating *FT* expression (Yanovsky and Kay, 2003). The phytochrome gene *PHYC* also contributes to variation in flowering time (Balasubramanian *et al.*, 2006), and the *PHYA* and *PHYB* genes are involved in the posttranscriptional regulation of CO and contribute to the regulation of the photoperiod pathway in *Arabidopsis* (Valverde *et al.*, 2004). In rice, *Heading date 3a* (*Hd3a*) was first detected as a quantitative trait locus that promotes flowering under short-day (SD) conditions. *Hd3a* encodes an orthologue of *Arabidopsis FT* (Ahn *et al.*, 2006). Recently, it has been shown in *Oryza sativa* that Hd3a/FT interacts with 14-3-3 proteins in shoot apical cells, yielding a complex that translocates to the nucleus and binds to the bZIP transcription factor (FD1), a rice homologue of *Arabidopsis thaliana* FD. The resultant ternary ‘florigen activation complex’ (FAC) induces transcription of the homologue of *Arabidopsis thaliana APETALA1* (*AP1*), the homologue of wheat *VRN1*, or rice *OsMADS15*, which leads to flowering (Taoka *et al.*, 2011).

In wheat, a mechanism was proposed where TaFT1 interacts with a bZIP transcription factor, *Triticum aestivum* FD-like 2 (TaFDL2), which has the ability to bind to the wheat *TmVRN1* promoter (Li and Dubcovsky, 2008). FT1 integrates the signals from the photoperiod pathway through its interactions with Photoperiod 1 (PPD1) and CO in temperate cereals (Turner *et al.*, 2005). Most of the natural variation in the response to photoperiod is concentrated in the *PPD1* locus (Beales *et al.*, 2007; Turner *et al.*, 2005; Wilhelm *et al.*, 2009), whereas variations in the response to vernalization occur in the *VRN1*, *VRN2*, and *VRN3* genes (Beales *et al.*, 2007; Distelfeld *et al.*, 2009; Trevaskis *et al.*, 2007). The timing of floral transition is associated with the heading time in cereal crops such as wheat and barley, and constitutes an important character because of its influence on adaptability to various environmental conditions. Bread wheat (*Triticum aestivum*, 2n=6x=42, genome constitution *AABBDD*) is grown in a wide range of environmental conditions and its wide adaptability results from the variation in heading time among cultivars. The genetic control of heading time in wheat has been clarified by many genetic studies, and three characteristics have been identified: vernalization requirement, photoperiod sensitivity, and narrow-sense earliness (Worland *et al.*, 2001). Wheat varieties are divided into winter and spring on the basis of their requirement of an extended period of cold to develop freezing tolerance and to flower (vernalization). The winter varieties that require vernalization to flower are more tolerant to freezing compared with spring varieties that do not require vernalization (Winfield *et al.*, 2010). In the winter varieties of bread wheat, vernalization is regulated by the vernalization genes *Vrn-A1*, *Vrn-B1*, and *Vrn-D1* located on chromosomes 5A, 5B, and 5D, respectively (Worland *et al.*, 2001). *Vrn-A1*, *Vrn-B1*, and *Vrn-D1* are all homeologs of the *Vrn-1* gene. Using a map-based method, *Vrn-A* ^*m*^ *1*, an orthologue of *Vrn-A1*, was cloned in diploid einkorn wheat *Triticum monococcum* (2n=2x=14, *A* ^*m*^ *A* ^*m*^), and the resulting gene was named *VRN1* (Yan *et al.*, 2003). In hexaploid wheat, it has been named *TaVRT-1* (*Triticum aestivum Vegetative to Reproductive Transition-1*) (Danyluk *et al.*, 2003) or *WAP1* (wheat *AP1*) (Murai *et al.*, 2003). Transgenic and mutant analyses in wheat revealed that *VRN1* has an indispensable role in the floral transition pathway (Loukoianov *et al.*, 2005; Murai *et al.*, 2003; Shitsukawa *et al.*, 2007). In addition to the genetic regulation of flowering, a hormonal involvement has also been reported. A cross-regulatory mechanism implicating abscisic acid, jasmonates, and auxin in the floral initiation process was reported in soybean (Wong *et al.*, 2009). Methyl jasmonate (MeJA) is a volatile compound initially identified from flowers of *Jasminum grandiflorum* and found to be distributed ubiquitously throughout the plant kingdom where it acts as a vital cellular regulator that mediates diverse developmental processes and defence responses against biotic and abiotic stresses (Cheong and Choi, 2003). In maize, MeJA was identified as an essential signal in determining the male floral structure on the tassel (Avanci *et al.*, 2010; Browse, 2009; Yu *et al.*, 2006). In *Pharbitis nil*, MeJA inhibited flowering in a photoperiodic-dependent mechanism, and high concentrations inhibited root and shoot growth (Maciejewska *et al.*, 2004; Maciejewska and Kopcewicz, 2003). MeJA treatment inhibited growth and flowering in *Chenopodium rubrum* plants under favourable growth conditions (Albrechtova and Ullmann, 1994). In *Arabidopsis*, a recent report showed that jasmonate caused a flowering delay and enhanced protection against biotic stress (Chehab *et al.*, 2012). However, there is no evidence of its implication in vernalization in temperate plants such as wheat.

The identification of a non-flowering cultivated diploid einkorn (*Triticum monococcum* ssp. *monococcum* L., 2*n=*2*x=*14, genome *A* ^*m*^ *A* ^*m*^) wheat mutant, *maintained vegetative phase*, which lacks the *VRN1* gene, provided a new tool to understand the function and regulation of VRN1. This mutant was induced by an ion-beam treatment that resulted in the *maintained vegetative phase* (*mvp*) phenotype and does not transit from the vegetative to the reproductive phase (Shitsukawa *et al.,* 2007). It was shown that the deleted region covers more than the *VRN1* gene and its promoter (Distelfeld and Dubcovsky, 2010) and may affect aspects of plant development other than flowering. In addition to *VRN1*, the *CYSTEINE PROTEINASE* gene (*TmCYS*) and *PHYTOCHROME C* gene (*TmPHYC*) were shown to be deleted in *mvp*-mutant plants (Distelfeld and Dubcovsky, 2010). The *Tm*CYS protein belongs to a family involved in a variety of proteolytic functions in plants, particularly those associated with processing and degradation of seed storage proteins and fruit ripening. Such proteins are also induced in response to other stresses such as wounding, cold, drought, programmed cell death, and senescence processes (Prins *et al.*, 2008). In temperate cereals, two MADS-box transcription factor *AP1-like FRUITFULL* (*FUL*) genes are similar to *VRN1* and are designated as *FUL2* (=HvMADS8=Os-MADS15) and *FUL3* (=HvMADS3=OsMADS18) (Preston and Kellogg, 2006). They were shown to be upregulated proportional to the duration of the cold treatment during vernalization, independently of *VRN1*, and are positively regulated by FT (Chen and Dubcovsky, 2012). The *TmPHYC* gene belongs to a family of red/far-red photoreceptors that includes the *PHYA* and *PHYB* genes (Devos *et al.*, 2005; Furuya, 1993). The association of the *PHYC* gene with the regulation of flowering initiation and the role of the phytochromes in light signalling suggest that the deletion of *TmPHYC* in the *mvp* mutant may have an effect on the regulation of the flowering promoter gene *TmFT1* (Distelfeld and Dubcovsky, 2010). A putative role of the *PHYC* gene in the downregulation of *FT1* expression is supported by the rapid response of these genes to light signals. When light conditions change from dark to light or from light to dark, a rapid change in *FT1* transcript level was observed (Distelfeld and Dubcovsky, 2010).

To determine the impact of the absence of *VRN1* and other deleted genes in the *mvp* mutant on global gene expression during cold exposure, we compared the transcriptome of the *mvp* mutant with that of wild-type plants using the Affymetrix GeneChip wheat genome array. The data obtained revealed that the *mvp* mutation caused the upregulation of pathogenesis-related (PR) genes related to the plant defence mechanism and jasmonate-responsive genes. These responses were associated with the accumulation of MeJA and cold-regulated proteins in the *mvp* mutant, suggesting a possible relationship between MeJA, cold responses, and flowering in wheat. Further studies using both hexaploid winter and spring wheat suggest that MeJA is involved in modulating vernalization and the floral transition.

## Materials and methods

### Plant material and growth conditions

Two spring wheat cultivars (*Triticum aestivum*, 2n=6x=42, AABBDD; Manitou and Bounty), one winter wheat cultivar (Norstar), wild-type einkorn wheat (*Triticum monococcum*, 2n=2x =14, *A* ^*m*^ *A* ^*m*^) and the *mvp*-mutant einkorn wheat were grown in a controlled growth chamber as previously described (Diallo *et al.*, 2010). Briefly, plants were grown at 20 ºC under long day (LD: 16h at 175 μmol m^–2^ s^–1^ and 8h dark) or short day (SD: 8h at 175 μmol m^–2^ s^–1^ and 16h dark) conditions as specified for each experiment. The einkorn wheat mutant, *mvp* (Shitsukawa *et al.*, 2007), and the control wild type of einkorn wheat were cold-acclimated for one week and used for both microarray analyses and MeJA quantification. The identification of heterozygous and homozygous *mvp*-mutant plants is described in the supporting information section.

### Microarray profiling and data analysis

The quality of RNA from the three biological replicates of wild-type and homozygous *mvp*-mutant plants was assessed on agarose gels and with the Bioanalyzer 2100 (Agilent). Microarray profiling was performed according to Affymetrix protocols at the Functional Genomics Platform of McGill University and Génome Québec Innovation Centre using the Affymetrix GeneChip® Wheat Genome Array. The microarray data were analysed using FlexArray software (1.6.1) that contains the robust multiarray average software used for data normalization. A two-fold cut-off value of expression and ANOVA analyses with a *P* value ≤0.05 were set to indicate differential gene expression between *mvp* and wild-type plants. Genes that were differentially expressed in *mvp*-mutant compared with wild-type plants were retained. Each gene was subjected to BLAST search against Genbank and Uniprot databases to obtain their Genbank accession number, UniProt or NCBI description. The microarray data were submitted to GEO and the accession number is GSE50882.

### Quantification of jasmonates

For *Triticum monococcum,* three-week-old plants of wild type and *mvp* mutant grown at 20 ºC under LD conditions were cold-acclimated at 4 ºC for one week. For winter wheat cv Norstar, two-week-old plants grown at 20 ºC under LD conditions were vernalized under SD conditions at 4 ºC for nine weeks and deacclimated for two weeks at 20 ºC under LD conditions. The whole aerial part of these plants were cut, and quickly frozen and ground with dry ice. A sufficient amount of plant material (500mg for each biological replicate) was used to analyse the jasmonates content in the different plants. Additional details are included in the supporting information section.

### Effects of MeJA on flowering time in wheat

To investigate the possible involvement of MeJA in the control of flowering time, spring wheat that does not require vernalization for flowering was treated with MeJA. Three-week-old diploid einkorn wheat plants (wild type) and hexaploid spring wheat plants (cv Manitou) grown at 20 ºC under LD conditions were sprayed with 150 μM MeJA dissolved in 0.1% tween 20 solution (treated plants) and with 0.1% tween 20 solution (control plants) every day for two weeks. The phenological development of the plants under different conditions was determined by measuring the final leaf number (FLN) as previously described (Wang *et al.*, 1995). The FLN on the main shoot of each plant at flag leaf stage was recorded. The number of flowering plants from control and treated plants was counted until all plants flowered. The percentage of plants with spikelets, emerged or not, for a given week was calculated. This experiment was repeated six times.

### Gene expression analysis

Three micrograms of total RNA was reverse-transcribed (RT) and an optimal amount was used for both RT-PCR (reverse transcription polymerase chain reaction) and qRT-PCR (quantitative RT-PCR) analyses. RT was performed using SuperScript II^TM^ First-Strand Synthesis System according to manufacturer’s instructions (Life Technologies). PCR analysis was performed using specific primers for each analysed gene. Real-time PCR analysis was performed in the Light Cycler 480 (Roche Applied Science) using the RT^2^ SYBR Green Master Mix (Qiagen) according to the manufacturer’s protocol. The detailed protocol for quantitative PCR, optimization conditions, thermocycling parameters, and data analysis are detailed in (Diallo *et al.*, 2012). Primers used in this study are listed in Supplementary Table S1 (available at *JXB* online).

## Results

### Transcriptome analysis of *mvp*-mutant plants using microarrays

**Fig. 1.**
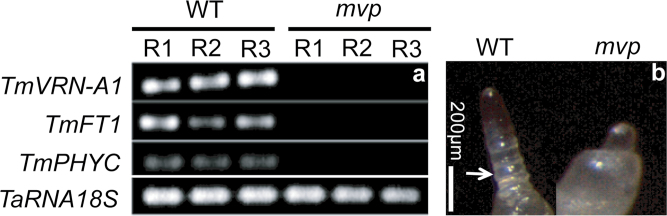
Molecular characterization and apex stage development of the *mvp* mutant. (a) Total RNA was extracted from whole aerial parts and analysed by RT-PCR. Plants were acclimated at 4 ºC for 7 d under LD conditions. Each replicate (R1, R2, and R3) was obtained from three control wild-type plants (WT) or from five mutant plants (*mvp*). Relative expression level of gene markers of wild-type and *mvp*-mutant plants was analysed by RT-PCR to validate the *mvp* mutant. The 18S *TaRNA* was used for load control. The experiments were repeated three times and a representative result is shown. (b) Apex stage development of wild-type (WT) and *mvp*-mutant plants (*mvp*) used for molecular characterization and microarray analysis. The arrow indicates the double ridge and the scale bar is indicated.

For the microarray study, three-week-old plants grown under LD conditions at 20 ºC were cold-acclimated at 4 ºC for one week under LD conditions for both wild-type einkorn spring wheat and *mvp*-mutant plants. The einkorn wheat is a spring habit wheat that is usually cultivated under LD conditions. This experiment was designed to compare global gene expression in both the wild type and *mvp* mutant, and to identify the genes affected by the deletion of *TaVRN1* and other genes in the *mvp*-mutant plants when grown at low temperature. The cold-acclimated plants were also used to evaluate freezing tolerance as flowering genes were proposed to influence the duration and level of freezing tolerance (Prášil *et al*., 2005). Homozygous *mvp*-mutant plants were identified by measuring the expression level of known markers for these plants: *TmVRN1*, *TmFT1* (Shimada *et al.*, 2009), and *TmPHYC* (Distelfeld and Dubcovsky, 2010). All marker genes were not expressed in the *mvp* plants, whereas their expression was normal in wild-type plants (Fig. 1a). The apex development of both wild-type and *mvp* plants are shown in Fig. 1b. It is clear that the wild-type apex on the left is highly extended and indicates the appearance of the double ridge, whereas the *mvp* apex on the right is still vegetative showing only the apical dome. These results confirm the developmental differences between the wild-type control plants, that are competent to flower, and the *mvp*-mutant plants that remain in the vegetative stage.

In addition to the non-flowering phenotype, plant size and number of emerged leaves were affected in *mvp*-mutant plants. Three weeks after germination, homozygous *mvp*-mutant plants are dwarf and have 2–3 leaves on the main shoot, whereas heterozygous *mvp*-mutant plants as well as wild-type plants have 4 leaves. The homozygous *mvp*-mutant plants continue to develop tillers without stem elongation, in contrast to the wild-type and *mvp* heterozygous plants. After three weeks, the wild-type and *mvp* heterozygous plants reach a maximum size ranging from 65–70cm, whereas the homozygous *mvp*-mutant plants do not exceed 7–8cm. It was also observed that root architecture is poorly developed in *mvp* plants compared with wild-type plants (Supplementary Fig. S1 available at *JXB* online).

**Fig. 2.**
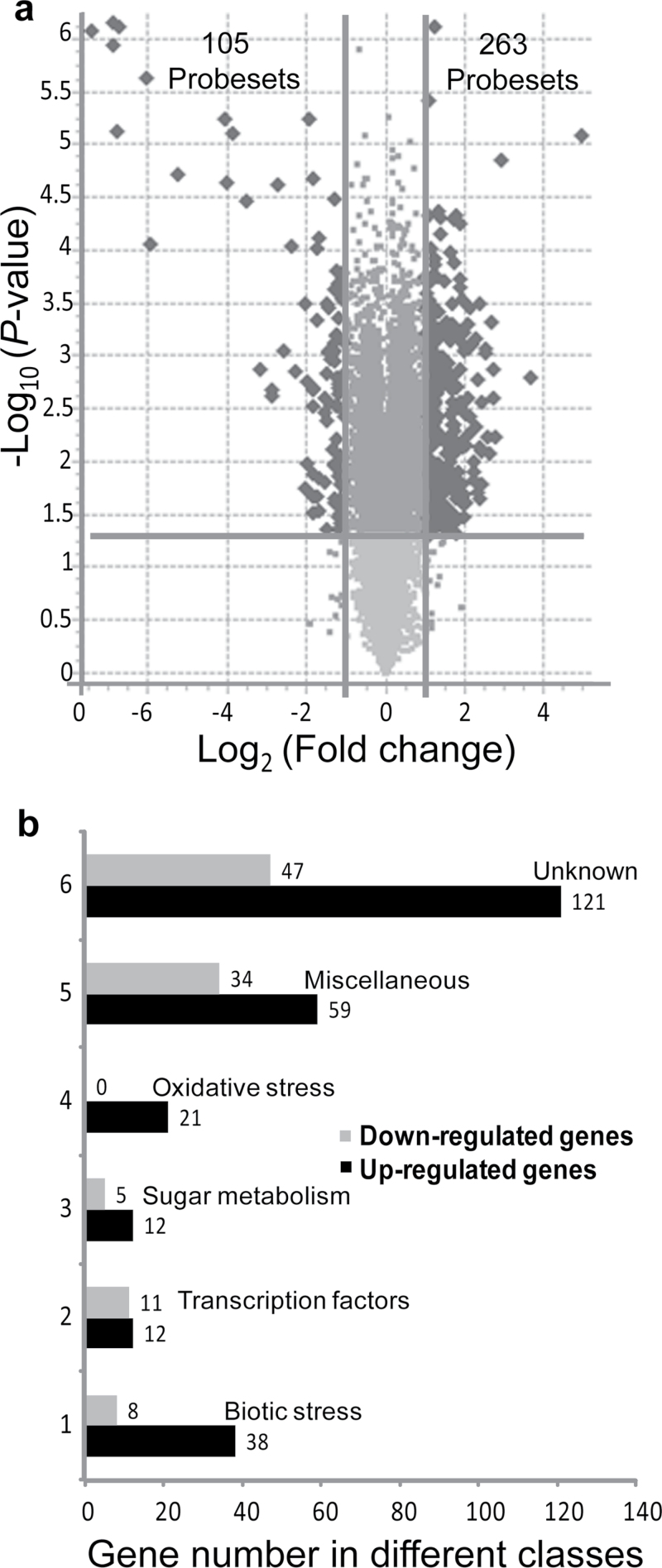
Microarray analysis of the *mvp*-mutant and the wild-type seedlings. (a) Volcano plots illustrating the log_2_ fold changes in gene expression differences between *mvp* mutants and wild-type plants. Probesets with statistically different expression (*P*≤0.05) and fold changes of ≥2-fold (263) are shown in the upper right corner and probesets with ≤–2-fold (105) are shown in the upper left corner. (b) Differentially regulated genes in *mvp*-mutant plants compared with wild-type plants derived from the microarray analysis: The differentially regulated genes were subdivided into six classes: Biotic-stress related genes (1), transcription factors (2), sugar metabolism (3), oxidative stress (4), miscellaneous genes (5), and unknown genes (6). The same RNA samples used in Fig. 1 were used for microarrays.

Cold-acclimated *mvp* and wild-type plants were analysed using microarrays to identify genes that are affected by the deletion of *VRN1* and other genes in the *mvp* mutant. The analysis showed that 368 probeset IDs (probeset or gene) were differentially expressed (cut-off ± 2-fold and ANOVA analyses with a *P*-value ≤0.05) between *mvp* and wild-type plants. These genes were subdivided in two groups: differentially upregulated (2-fold and up, 263 genes) and differentially downregulated genes (–2-fold and down, 105 genes; Fig. 2a). This represents less than 1% of the total genes on the microarray (61,290) indicating that most of the genes are expressed at a similar level between the two types of plants. The 263 upregulated genes can be distributed in six major classes and the 105 downregulated genes in five major classes (Fig. 2b) based on gene ontology search. These classes are: biotic-stress-related genes (Table 1), transcription factors (Supplementary Table S2 available at *JXB* online), sugar-metabolism-related genes (Supplementary Table S3 available at *JXB* online), oxidative-stress-related genes (Supplementary Table S4 available at *JXB* online), miscellaneous genes (Supplementary Table S5 available at *JXB* online), and unknown genes (Supplementary Table S6 available at *JXB* online). Miscellaneous genes regroup known genes scattered in small numbers in various other classes based on gene ontology. The unknown class consists of genes that do not have a clear function. Although several gene sequences from this class may have known domains, it would be highly speculative to assign them a specific function based on these broadly defined domains.

**Table 1.** *mvp* wheat biotic stress regulated genes identified by microarray

Affymetrix probesets ID	GenBank accession	UniProt and NCBI description	Fold change
Ta.3976.2.S1_x_at	CA679100	*Triticum aestivum* flavanone 3-hydroxylase mRNA, partial cds	6.713
Ta.18574.1.A1_x_at	CK196896	Ice recrystallization inhibition protein 4 (Fragment) *n*=1 Tax=*Triticum aestivum* RepID=B9VR51_WHEAT	5.807
Ta.3976.1.S1_at	CA678526	*Triticum aestivum* flavanone 3-hydroxylase mRNA, partial cds	5.510
Ta.27327.1.S1_x_at	BT009360	Pathogenesis-related 1a *n*=1 Tax=*Triticum monococcum* RepID=Q3S4I4_TRIMO	5.467
Ta.959.1.S1_at	CA721939	Thaumatin-like protein *n*=3 Tax=*Triticum* RepID=Q41584_WHEAT	5.147
Ta.221.1.S1_at	AF112963	Chitinase II *n*=1 Tax=*Triticum aestivum* RepID=Q9XEN3_WHEAT	4.725
Ta.27762.1.S1_x_at	AF384146	Pathogenesis-related protein 1A/1B *n*=10 Tax=*Triticeae* RepID=PR1A_HORVU	4.714
Ta.24501.1.S1_at	CD863039	Pathogenesis-related protein 1A/1B *n*=10 Tax=*Triticeae* RepID=PR1A_HORVU	4.689
Ta.22619.1.S1_at	CA687670	Pathogenesis-related protein 10 *n*=1 Tax=*Hordeum vulgare* RepID=Q84QC7_HORVU	4.186
Ta.22619.1.S1_x_at	CA687670	Pathogenesis-related protein 10 *n*=1 Tax=*Hordeum vulgare* RepID=Q84QC7_HORVU	4.053
TaAffx.104812.1.S1_s_at	BJ223744	Lipoxygenase 2.1, chloroplastic *n*=1 Tax=*Hordeum vulgare* RepID=LOX21_HORVU	4.031
TaAffx.28302.2.S1_at	CA695754	Dirigent-like protein *n*=2 Tax=*Oryza sativa* RepID=Q53NP6_ORYSJ	4.006
Ta.1967.1.S1_x_at	CK152466	Lipoxygenase 2.1, chloroplastic *n*=1 Tax=*Hordeum vulgare* RepID=LOX21_HORVU	3.927
Ta.1967.2.A1_x_at	AJ614579	Lipoxygenase 2.1, chloroplastic *n*=1 Tax=*Hordeum vulgare* RepID=LOX21_HORVU	3.691
Ta.23322.2.S1_at	CA640491	Thaumatin-like protein TLP8 *n*=1 Tax=*Hordeum vulgare* RepID=Q946Y8_HORVU	3.672
Ta.224.1.S1_at	AF112966	Chitinase IV *n*=1 Tax=*Triticum aestivum* RepID=Q9XEN6_WHEAT	3.375
TaAffx.107480.1.S1_at	CA679967	Ice recrystallization inhibition protein 5 *n*=1 Tax=*Deschampsia antarctica* RepID=C0L702_DESAN	3.357
TaAffx.128595.1.S1_at	CK216241	Putative vacuolar defence protein *n*=2 Tax=*Triticum aestivum* RepID=Q6PWL8_WHEAT	3.294
Ta.2709.1.S1_s_at	CK166154	Defensin-like protein 2 *n*=1 Tax=*Triticum aestivum* RepID=DEF2_WHEAT	3.188
Ta.30921.1.S1_x_at	CN012317	12-oxo-phytodienoic acid reductase *n*=1 Tax=*Zea mays* RepID=Q49HE1_MAIZE	2.948
Ta.7022.1.S1_s_at	BJ281221	Phenylalanine ammonia-lyase *n*=5 Tax=Poaceae RepID=Q7F929_ORYSJ	2.868
TaAffx.137429.1.S1_at	CA610138	Dehydrin 5 (Fragment) *n*=1 Tax=*Hordeum vulgare* subsp. spontaneum RepID=Q6V7D2_HORSP	2.645
Ta.7022.2.S1_at	BF199967	Phenylalanine ammonia-lyase *n*=1 Tax=*Triticum aestivum* RepID=PALY_WHEAT	2.604
Ta.25077.1.A1_at	BQ161103	Ice recrystallization inhibition protein 2 *n*=1 Tax=*Triticum aestivum* RepID=Q56B89_WHEAT	2.580
Ta.7022.2.S1_x_at	BF199967	Phenylalanine ammonia-lyase *n*=1 Tax=*Triticum aestivum* RepID=PALY_WHEAT	2.569
Ta.21556.1.S1_at	CA684533	Protein WIR1B *n*=1 Tax=*Triticum aestivum* RepID=WIR1B_WHEAT	2.505
Ta.25026.1.S1_at	BQ804965	Dehydrin *n*=1 Tax=*Triticum turgidum* subsp. durum RepID=Q5CAQ2_TRITU	2.504
Ta.21768.1.S1_x_at	CA701727	Ice recrystallization inhibition protein 7 *n*=1 Tax=*Deschampsia antarctica* RepID=C0L704_DESAN	2.475
Ta.16472.1.S1_s_at	CA606887	Pathogenesis-related protein n=1 Tax=*Hordeum vulgare* RepID=P93181_HORVU	2.418
Ta.12663.1.S1_at	CK197682	Ice recrystallization inhibition protein 1 n=1 Tax=*Triticum aestivum* RepID=Q56B90_WHEAT	2.334
TaAffx.128418.43.S1_at	BJ252866	Endochitinase *n*=5 Tax=Pooideae RepID=Q41539_WHEAT	2.324
Ta.28659.3.S1_x_at	CA689419	Putative protease inhibitor n=1 Tax=*Hordeum vulgare* RepID=Q96465_HORVU	2.317
Ta.22678.1.A1_a_at	CK214868	Chitinase 1 *n*=2 Tax=Andropogoneae RepID=B4FBN8_MAIZE	2.294
TaAffx.45277.1.S1_x_at	BJ231180	Phenylalanine ammonia-lyase *n*=1 Tax=*Triticum aestivum* RepID=PALY_WHEAT	2.263
Ta.2278.2.S1_a_at	CK196331	Chitinase IV *n*=1 Tax=*Triticum aestivum* RepID=Q9XEN6_WHEAT	2.254
Ta.2278.3.S1_x_at	CD490414	Chitinase II *n*=1 Tax=*Triticum aestivum* RepID=Q9XEN3_WHEAT	2.234
Ta.27389.2.S1_x_at	BJ297034	Defensin-like protein 2 *n*=1 Tax=*Triticum aestivum* RepID=DEF2_WHEAT	2.202
Ta.2278.2.S1_x_at	CK196331	Chitinase IV *n*=1 Tax=*Triticum aestivum* RepID=Q9XEN6_WHEAT	2.086
Ta.12820.1.S1_at	CK215415	Defensin *n*=1 Tax=*Triticum turgidum* subsp. durum RepID=C9E1C6_TRITU	-2.245
Ta.28133.1.A1_s_at	CA636835	Dirigent-like protein, expressed *n*=2 Tax=*Oryza sativa* RepID=Q2R0I1_ORYSJ	-2.438
Ta.7963.2.S1_x_at	CK215257	Dirigent-like protein, expressed *n*=2 Tax=*Oryza sativa* RepID=Q2R0I1_ORYSJ	-2.584
Ta.7388.2.S1_x_at	BU672305	Jasmonate-induced protein *n*=2 Tax=*Triticum aestivum* RepID=A7LM74_WHEAT	-2.636
Ta.7108.1.S1_at	CF134173	Ice recrystallization inhibition protein 2 *n*=1 Tax=*Triticum aestivum*	-3.427
TaAffx.98064.1.A1_at	BQ168859	Ice recrystallization inhibition protein 3 *n*=1 Tax=*Deschampsia antarctica*	-4.022
Ta.7388.1.S1_at	BJ320233	Jasmonate-induced protein *n*=2 Tax=*Triticum aestivum* RepID=A7LM74_WHEAT	-7.187
Ta.7388.2.S1_a_at	BU672305	Jasmonate-induced protein *n*=2 Tax=*Triticum aestivum* RepID=A7LM74_WHEAT	-7.230

The annotation is made according to Affymetrix Gene Chip® wheat genome array of the 46 differentially regulated biotic stress probeset IDs complemented with BLAST results showing the Genbank accession number, UniProt or NCBI description and is presented in decreasing order of differential expression of ≥2-fold and ≤–2-fold cut off.

The first class of genes (46 biotic stress genes) represents 12.5% of the differentially regulated genes (Fig. 2b). Most genes of this class are upregulated. Among them are genes encoding flavanone hydroxylase (increased 6.7-fold), ice recrystallization inhibition (5.8-fold), pathogenesis related 1a (5.4-fold), thaumatin (5.1-fold) chitinase (4.7-fold), lipoxygenase (4-fold), vacuolar defence protein (3.3-fold), defensin-like protein (3.2-fold), 12-oxo-phytodienoic acid reductase (2.9-fold), dehydrin (2.4-fold), and endochitinase (2.3-fold) (Table 1). The two most downregulated gene in this class are annotated as jasmonate-induced proteins (–7.2-fold). This annotation was based on their homology to *JRP-32*, which was first detected in jasmonate-treated leaves of barley. However, in wheat, the expression of *Ta-JA1* mRNA, which is the closest homologue, was confined to stem tissues, and was not detected in leaves even after treatment with jasmonate (Wang and Ma, 2005) suggesting that it is not regulated by jasmonate in wheat. Transcription factors represent 6.2% of the differentially regulated genes (23 genes out of 368 genes). This class contains genes encoding MADS-box, WRKY, and CBF transcription factors. The majority of genes that encode MADS-box transcription factors were downregulated, whereas those encoding CBF and WRKY transcription factors were upregulated (Supplementary Table S2 available at *JXB* online). The sugar-metabolism-related genes class contains 17 upregulated genes. This class encodes various enzymes including glucomannan 4-beta-mannosyltransferase 1 (6.8-fold), beta-glucanase (6.6-fold), glucan endo-1,3-beta-glucosidase (3.9-fold), UDP-glucosyl transferase (3.4-fold) and glucan endo-1,3-beta-glucosidase (3.4-fold) (Supplementary Table S3 available at *JXB* online). The fourth class represents 21 oxidative-stress-related genes that are all upregulated. This class contains genes that encode cytochrome c oxidase (up by 3.8-fold), leucoanthocyanidin dioxygenase (3.3-fold), peroxidase (3.0-fold) cytochrome P450 (2.7-fold), and chloroplast lipocalin (2.4-fold) (Supplementary Table S4 available at *JXB* online). The miscellaneous genes class contains 93 genes (25.3%). These are involved in many aspects of plant development. Genes that are highly upregulated from this class include apyrase, chaperone protein, agmatine coumaroyltransferase, BRASSINOSTEROID INSENSITIVE (BRI) 1-associated receptor kinase 1, cold acclimation-associated protein WCOR518, and serine/threonine kinase-like protein (Supplementary Table S5 available at *JXB* online). Multiple aspects of plant growth and development such as expression of a variety of developmental programmes including cell elongation, vascular differentiation, seed germination, senescence, and fertility are regulated by brassinosteroids (BRs) and require an active BRI1 receptor for hormone perception and signal transduction (Ehsan *et al.*, 2005). An important part of the differentially regulated genes have unknown function representing 168 out of the 368 genes (45.5%, in Supplementary Table S6 available at *JXB* online).

Taken together, these results suggest that the *mvp* mutation not only results in a non-flowering phenotype, but also activates the genes involved in the regulation of jasmonate biosynthesis and biotic stress responses.

### Validation of the microarray results

**Fig. 3.**
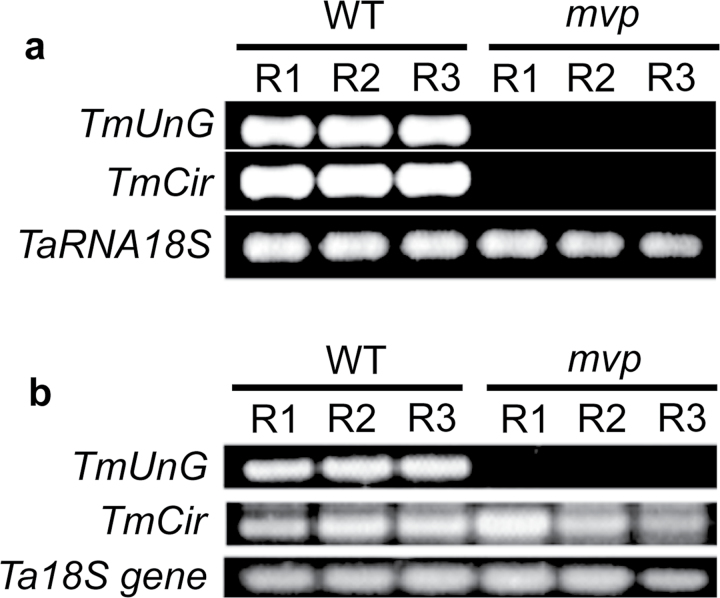
Relative expression level of several selected genes in *mvp*-mutant and wild-type control plants analysed by RT-PCR and PCR experiments. (a) Validation of *TmUnG* and *TmCir* expression by RT-PCR. The RNA samples are the same as those used in Fig. 1 and the microarray experiment. The 18S *TaRNA* was used as load control. (b) Genomic DNA was extracted from the same plant samples used for the microarray analysis and were tested for PCR amplification to analyse the possible deletion of the two most repressed genes from the array analysis (*TmUnG* repressed 114 times and *TmCir* repressed 169 times). The 18S DNA is used as control. The experiments were repeated with 3 different biological replicates with the same result. Each replicate (R1, R2, and R3) was obtained from five mutants plants (*mvp*) and from three control wild-type plants.

The most repressed gene (169-fold) encodes a protein similar to the circumsporozoite protein (*Tm*Cir). The second most repressed gene (*TmUnG* for *Triticum monococcum Uncharacterized Gene*) (114-fold) encodes an ‘Uncharacterized’ protein. To confirm the repression of these two genes, we measured the accumulation of their transcript level using RT-PCR. The results show that the *TmCir* and *TmUnG* are not expressed in the *mvp*-mutant plants compared with wild-type plants (Fig. 3a). To determine whether these two genes are not deleted in the *mvp*-mutant plants, we extracted genomic DNA from the same plant samples used for the microarray analysis to amplify the two genes by PCR. The results show that the coding region of the *TmCir* gene is not deleted in the *mvp* mutant. On the other hand, the *TmUnG* gene appears to be deleted in the *mvp*-mutant plants (Fig. 3b).

Expression analysis using RT-PCR (Fig. 1; data not shown for *TmCir*) and qRT-PCR (Supplementary Fig. S2a available at *JXB* online) confirmed the results from the microarray analysis of *TmVRN1*, *TmFT1*, *TmCir* and *TmUnG* showing very low or no detectable expression of these genes in *mvp*-mutant plants in comparison to wild-type plants, whereas a gene used as control (*TmGI*) showed a similar expression in both wild-type and *mvp*-mutant plants (Supplementary Fig. S2a available at *JXB* online). We also found a low expression level of *TmPHYC* in *mvp* plants compared with wild-type plants (Supplementary Fig. S2a available at *JXB* online and Table 2). The qRT-PCR analysis also showed a strong downregulation of *TmCYS* expression in *mvp* plants and its possible deletion as previously reported (Distelfeld and Dubcovsky, 2010). BLAST search of *TmCYS* gene from Affymetrix database of the wheat array reveals that *TmCYS* is absent in the wheat array. This explains why we did not find *TmCYS* among the 368 genes that are differentially regulated in the microarray analysis.

**Table 2.** *mvp* wheat plant genes validated by qRT-PCR and their fold change on the microarray

Affymetrix Probeset IDs	GenBank Accession	UniProt and NCBI description	Fold change
Ta.3976.2.S1_x_at	CA679100	*Triticum aestivum* flavanone 3-hydroxylase mRNA, partial cds	6.713
Ta.27327.1.S1_x_at	BT009360	Pathogenesis-related 1a *n*=1 Tax=*Triticum monococcum* RepID=Q3S4I4_TRIMO	5.467
Ta.959.1.S1_at	CA721939	Thaumatin-like protein *n*=3 Tax=*Triticum* RepID=Q41584_WHEAT	5.147
Ta.221.1.S1_at	AF112963	Chitinase II *n*=1 Tax=*Triticum aestivum* RepID=Q9XEN3_WHEAT	4.725
TaAffx.104812.1.S1_s_at	BJ223744	Lipoxygenase 2.1, chloroplastic *n*=1 Tax=*Hordeum vulgare* RepID=LOX21_HORVU	4.031
Ta.10215.1.S1_at	AY679115	*Triticum aestivum* gigantean 3 (TaGI3) mRNA, complete cds	–0.035
Ta.11017.1_A1_at	B4ERX7	*Triticum aestivum O*-methyltransferase	–0.447
Ta.30640.1.S1_at	CD861747	*Triticum aestivum* flowering locus T mRNA, complete cds or VRN3	–3.250
Ta.28005.1.A1_at	CD862101	Phytochrome C (Fragment) *n*=1 Tax=*Hordeum vulgare* RepID=Q945T7_HORVU	–16.267
TaAffx.143995.17.S1_s_at	AY188331	MADS box transcription factor *n*=15 Tax=*Triticeae* RepID=O82128_WHEAT	–108.744
TaAffx.85922.1.S1_s_at	CA618396	Putative uncharacterized protein Sb01g007930 n=2 Tax=Poaceae RepID=C5X0B2_SORBI (UnG)	–114.866
Ta.29481.1.S1_at	CK194207	Circumsporozoite protein *n*=2 Tax=Andropogoneae RepID=B6TF33_MAIZE	–169.064

The annotation is made according to Affymetrix Gene Chip® wheat genome array of the 12 probeset IDs used in RT-PCR and qRT-PCR experiments to validate microarray expression profiling analysis complemented with BLAST results showing the Genbank accession number, UniProt or NCBI description and is presented in decreasing order of differential expression of ≥2-fold and ≤–2-fold cut off.

To further validate the microarray analysis, we selected several genes that are upregulated in the *mvp* plants and another control gene showing a similar expression in wild-type and *mvp*-mutant plants (*TmOMT*). The five upregulated genes *TmTha* (*thaumatin*)*, TmChi* (*chitinase*), *TmLox2* (*lipoxygenase 2*), *TmFlav* (*flavanone hydroxylase*) and *TmPR1a* (*pathogenesis related 1a*) are all associated with biotic stress (Supplementary Fig. S2b, available at *JXB* online, and listed in Table 1). *Lox2* encodes the enzyme that catalyses an important step of the biosynthesis of jasmonic acid (JA) from membrane-derived linolenic acid (Beale and Ward, 1998; Farmer *et al.*, 2003). Genes encoding LOX2 and FLAV are known to be highly induced by jasmonates (JA and MeJA) or in response to biotic stress (Edreva, 2005), whereas the gene encoding PR1a is a salicylic acid responsive gene (Jaulneau *et al.*, 2010; Mur *et al.*, 2006). PR1 proteins are defence factors ubiquitously synthesized by plants in response to pathogen infections. On the other hand, chitinases, 1,3-beta-glucanases, thaumatin-like proteins and peroxidases are involved in growth, development and defence processes (Sabater-Jara *et al.*, 2011). These proteins contribute, directly or indirectly, to resistance to pathogen attack together with other defence proteins, such as 1,3-beta-glucanases, chitinases, and secondary metabolism enzymes including phytoalexin biosynthetic enzymes (Rivière *et al.*, 2008). The microarray analysis shows a high expression level of these five genes (*TmTha, TmChi, TmLox2*, *TmFlav* and *TmPR1a*) in *mvp* plants compared with wild-type plants (Table 1). The qRT-PCR analysis of these five upregulated genes confirms the expression levels observed in the microarray analysis (Supplementary Fig. S2b available at *JXB* online). Overall, RT-PCR and qRT-PCR analyses of twelve genes (2 control similarly regulated genes, 5 downregulated genes and 5 upregulated genes) confirm the microarray results demonstrating the reliability of the global transcriptome analysis (Supplementary Fig. S2 available at *JXB* online). The microarray data, the Affymetrix probeset and the description of these genes are presented in Table 2. As controls, we used *TmGI* (accession number AY679115), a photoperiodic gene implicated in the flowering process and *TmOMT* (accession number B4ERX7) encoding an *O*-methyltransferase. Their expression did not vary in the qRT-PCR analysis between *mvp*-mutant and wild-type plants (Supplementary Fig. S2, available at *JXB* online, and Table 2). Nucleotide sequence alignment between *TmOMT* (B4ERX7) used as control and the upregulated *TaOMT* (3.3-fold, accession number AJ614654 in Supplementary Table S5 available at *JXB* online) showed no significant similarity indicating that these two *O*-methyltranferases are different. Taken together, the microarray analysis showed that the *mvp* mutant upregulates the expression of defence-related genes known to be induced by pathogen attack, jasmonates and cold, whereas it represses the flowering genes in comparison to wild-type plants under the same growth conditions.

### Freezing tolerance and COR proteins accumulation in *mvp* plants

To test whether the *maintained vegetative phase* is associated with enhanced freezing tolerance, freezing tests were performed in cold-acclimated and non-acclimated homozygous and heterozygous *mvp*-mutant and wild-type plants. Non-acclimated plants from the three types of plants died after freezing at –6 ºC, whereas all the cold-acclimated plants survived at this temperature.

Freezing tests of cold-acclimated plants at –8 ºC showed that homozygous and heterozygous mutants have higher survival rates (79.2% and 60.4%, respectively) compared with the wild-type plants (45.8%). Together, these results indicate that the homozygous *mvp*-mutant plants are more freezing tolerant compared with the heterozygous and wild-type plants. To support the freezing tests results, we evaluated the expression of different COR proteins using immunoblot analysis. The analysis shows a higher accumulation of two major COR proteins, WCS120 and WCS19, in the homozygous *mvp*-mutant plants compared with wild-type plants (Supplementary Fig. S3 available at *JXB* online). This is consistent with the improvement of freezing tolerance of *mvp*-mutant plants. These results suggest that the deletion of *VRN1* and other genes in einkorn *mvp*-mutant wheat enhances freezing tolerance and the accumulation of some COR proteins associated with higher freezing tolerance, compared with wild-type plants.

### Jasmonates content in *mvp* and wild-type einkorn wheat plants

Microarray results showed the upregulation of genes involved in jasmonate biosynthesis (*Lipoxygenase* and *12-oxo-phytodienoic acid reductase*) and of other genes associated with biotic stress. These observations suggest that *mvp* plants may synthesize more jasmonate than wild-type plants to induce the accumulation of PR genes. To test this hypothesis, the content of JA and MeJA was measured in *mvp*-mutant and wild-type einkorn wheat plants. New plant material prepared under the same conditions as the microarray experiment was used to analyse the hormonal content. A comparable quantity of JA was found in wild-type plants (148±7ng g^–1^ FW) and in *mvp*-mutant plants (149±4.57ng g^–1^ FW). On the other hand, MeJA was much higher in *mvp*-mutant plants (72.7±3.5ng g^–1^ FW) compared with wild-type plants (11.5±2.3ng g^–1^ FW). These results show that similar JA content is found in the two types of plants, whereas MeJA, the active hormone, accumulates more than 6-fold in *mvp* plants compared with wild-type einkorn wheat plants.

### MeJA levels during vernalization in hexaploid winter wheat

**Fig. 4.**
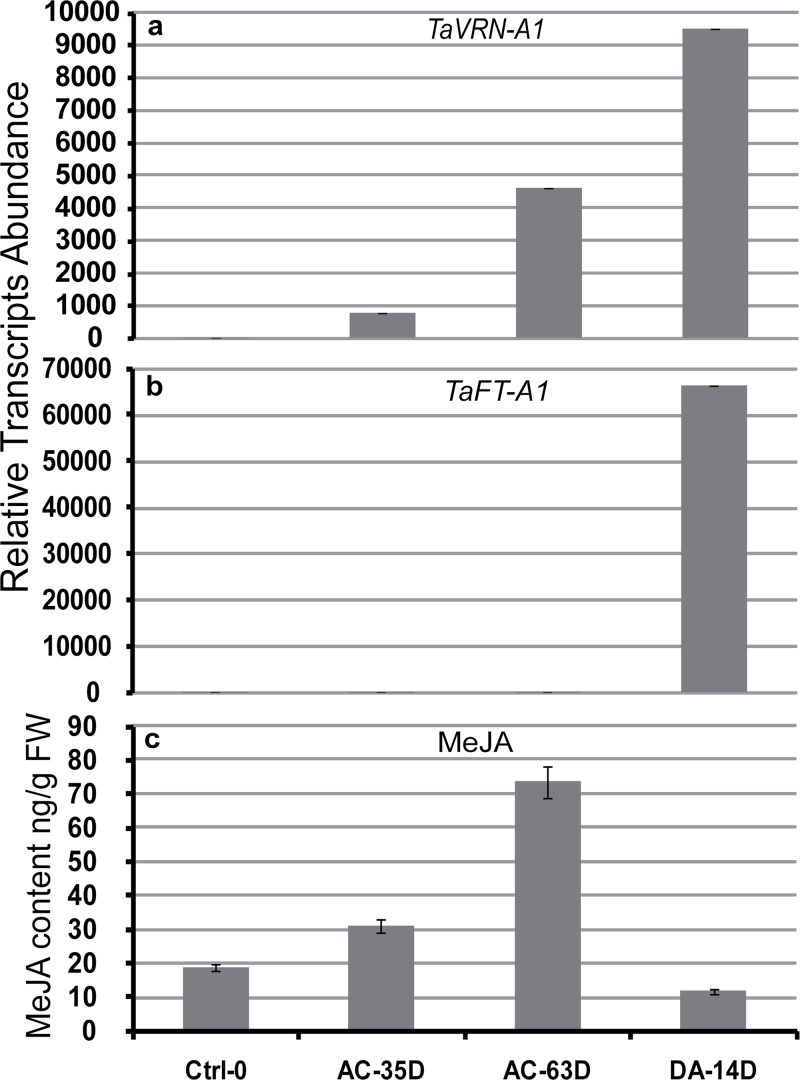
Relative expression level of *TaVRN1* and *TaFT1* and MeJA content during vernalization in hexaploid winter wheat seedlings analysed by qRT-PCR and HPLC/MS. Two weeks after germination at 20 ºC under LD conditions, non-vernalized winter wheat (cv Norstar) plants were vernalized under SD conditions at 4 ºC for 63 d and deacclimated for 14 d at 20 ºC under LD conditions. The aerial part was sampled around 4h after the beginning of the daylight period. The expression level of *TaVRN-A1* (panel a) and *TaFT-A1* (panel b) are expressed relative to the non-vernalized point (Ctrl-0). Data represent the mean ± SEM from three biological replicates. The expression levels are normalized with the *TaRNA 18S*. Plant samples from the same experiment were used to measure the MeJA content by HPLC / MS (panel c). Data represent the mean ± SEM from three biological replicates. AC-35D: cold-acclimated for 35 d; AC-63D: cold-acclimated for 63 d; DA-14D; plants cold-acclimated for 63 d and deacclimated under favourable growth conditions (LD and 20 ºC).

In *mvp* plants, the non-flowering phenotype is probably associated with the deletion of *VRN1* and *PHYC* and to the downregulation of *FT1* genes because of their importance in flowering regulation in wheat. The downregulation of *FT1* and the accumulation of MeJA in cold-acclimated *mvp* mutants suggest that MeJA levels may also be associated with the regulation of *TaVRN1* and *FT1* in wheat during cold acclimation/vernalization. Promoter analysis of both genes revealed that *TaVRN1* and *TaFT1* promoters contain one *cis*-acting regulatory element involved in MeJA-responsiveness (CGTCA-motif) (Diallo *et al.*, 2012). Together, these results suggest a role for MeJA during vernalization in wheat. To investigate this possibility, the MeJA content was measured in hexaploid winter wheat leaves during and after vernalization. Our data show a typical expression profile of both *TaVRN1* and *TaFT1* during vernalization of wheat plants (Fig. 4a, b). MeJA accumulated in response to cold and reached its maximal accumulation once vernalization is completed after 63 d, and then decreased when plants were transferred to inductive flowering conditions (Fig. 4c). At this stage, *TaVRN1* expression reached its highest level and MeJA was down to its basal level. We also determined the expression profile of *TaLox2*, *TmFlav*, *TmTha*, and *TmChi* (Supplementary Fig. S4 available at *JXB* online), which are known to be associated with jasmonate responses using the same vernalized and deacclimated samples. Results show that MeJA accumulation occurs in parallel to the expression kinetic of the four genes (Fig. 4c and Supplementary S4 available at *JXB* online). Together, these results indicate that MeJA accumulation may elicit the defence response needed to protect plants from pathogen damage during the sensitive phase, and thus allow plants to transit successfully from the vegetative phase to the reproductive phase when favourable growth conditions are encountered.

### Effect of MeJA on flowering

To test the hypothesis that high levels of MeJA can affect flowering time in vernalization-insensitive cultivars, three-week-old plants of two spring cultivars of hexaploid wheat (Manitou and Bounty) and the wild-type einkorn were treated with different concentrations of MeJA (100, 150, 200, 300, and 450 μM) at normal growth temperature (data not shown). The efficacy of the treatment was assessed by measuring the accumulation of jasmonates in the cv Manitou plants treated with 450 μM MeJA compared with control plants. The analysis indicates that the treated plants accumulate 805±117ng g^–1^ FW of MeJA compared with 120.6±11ng g^–1^ FW for control plants. The JA content was 221±4.9ng g^–1^ FW for treated plants and 28±1.4ng g^–1^ FW for non-treated plants. This result indicates that the level of jasmonates in treated plants is approximately 6-fold the level of jasmonates in non-treated plants, confirming the efficiency of the treatment. Plants from the three groups of plants treated with 100, 150 and 200 μM MeJA appeared healthy and showed delays in growth and flowering. Furthermore, plants treated with 100 μM of MeJA showed a less significant delay in both growth and flowering compared with 150 and 200 μM of MeJA. No significant difference was observed between the effects of 150 μM and 200 μM of MeJA. The plants treated with the two highest MeJA concentrations (300 and 450 μM) appeared less healthy and showed severe delay in growth and flowering, leaf senescence, and spikelets abnormalities. We thus used 150 μM of MeJA for further experiments with the wild-type einkorn wheat and the hexaploid spring wheat cultivar Manitou.

**Fig. 5.**
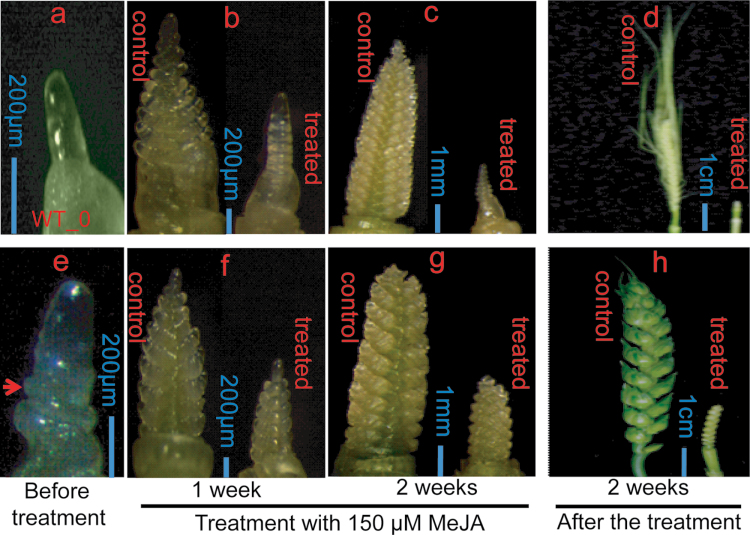
Effect of MeJA treatment on apex development in wild-type einkorn wheat (a–d) and spring wheat cv Manitou (e–h). Control and treated plants were grown at 20 ºC under long-day photoperiod (LD) conditions. Pictures of apex from dissected plants were taken before, during, and two weeks after MeJA treatment. (a, e) Apex of three-week-old wild-type einkorn wheat (a) or spring wheat Manitou (e) before treatment. (b–h) Control plants (sprayed with 0.1% tween 20 solution only) and treated plants (sprayed with 150 μM MeJA dissolved in 0.1% tween 20) were sprayed every day for one week (b, f) or two weeks (c, g); plants were then kept under the same growth conditions and apex pictures were taken two weeks (d, h) after the treatment. The scale bars are shown for each picture.

**Fig. 6.**
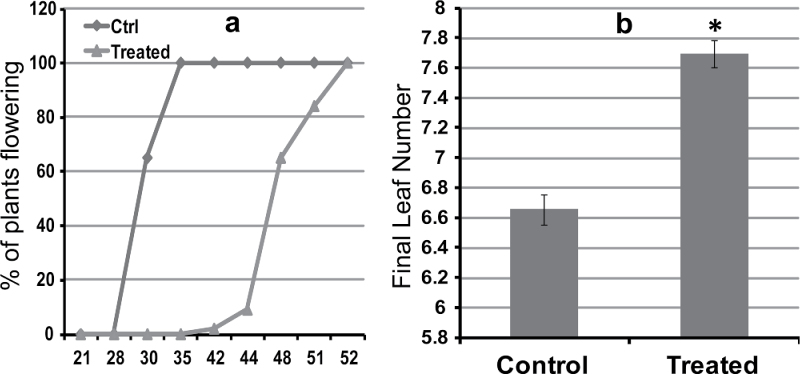
Effect of MeJA treatment on flowering and final leaf number in *Triticum aestivum* wheat plants cv Manitou. (a) Time course of flowering. Three-week-old plants were sprayed with 150 μM of MeJA dissolved in 0.1% tween 20 every day for 2 weeks at 20 ºC under LD conditions. Control plants were treated with 0.1% tween 20 solution only under the same growth conditions. The percentage of flowering plants was determined for both treated and control plants. (b) Final leaf number. Results were expressed as the mean ± SEM of six different experiments. Comparison between groups and analysis for differences between means of control and treated plants were performed using ANOVA followed by the post-hoc test Newman–Keuls. The threshold for statistical significance was: *: *P*<0.05.

The effect of MeJA treatment on flowering time was investigated by observing apex and plants development before, during, and two weeks after MeJA treatment (Fig. 5, Supplementary Figs S5 and S6, available at *JXB* online). Three-week-old spring diploid einkorn wheat (Fig. 5a–d and Supplementary Fig. S5b–d, available at *JXB* online) and hexaploid wheat cv Manitou plants (Fig. 5e–h and Supplementary Fig. S6b–d, available at *JXB* online) were separated into two groups; the control group was sprayed with 0.1% tween 20 (control plants) and the other group was sprayed with 150 μM MeJA, dissolved in 0.1% tween 20, every day for a period of 14 d (treated plants). The results showed that MeJA treatment reduced stem elongation, slowed apex differentiation, and delayed flowering of diploid einkorn wheat at the whole plant level (Fig. 5b–d and Supplementary Fig. S5c, d, available at *JXB* online). A similar result was obtained in the hexaploid spring wheat cv Manitou at the apex and whole plant level (Fig. 5f–h and Supplementary Fig. S6c, d, available at *JXB* online). The flowering delay, the reduction of stem elongation and the slow differentiation of apex were more evident in diploid einkorn wheat compared with hexaploid wheat. In addition to shoot growth inhibition, MeJA treatment reduced root growth as observed in *mvp* plants (Supplementary Fig. S1 available at *JXB* online), but did not prevent the emergence of new leaves (Fig. 6b). Spring wheat Manitou treated plants produced 7.7 leaves compared with non-treated plants that produced 6.6 leaves at the time of flowering (FLN measurements; Fig. 6b), suggesting that treated wheat plants remained in the vegetative phase for a longer period. The percentage of flowering plants was calculated for MeJA-treated and control non-treated plants (Fig. 6a). Flowering of treated plants was delayed by at least 14 d. In control plants, 65% and 100% of the plants flowered after 28 and 35 d, respectively, whereas none of the MeJA treated plants flowered at this time. About 4% of the MeJA treated plants flowered after 42 d, whereas all plants flowered after 51 d (Fig. 6a).

**Fig. 7.**
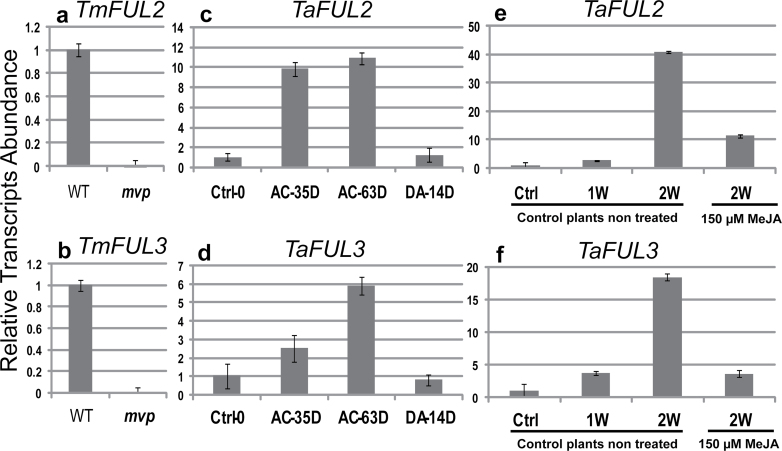
Relative expression level of *FUL2* and *FUL3* analysed by qRT-PCR. (a, b) *mvp*-mutant and wild-type einkorn wheat plants. Plants were acclimated at 4 ºC for 7 d under LD conditions. The data represent the mean obtained from three biological replicates each using three control wild-type plants (WT) or five *mvp*-mutant plants (*mvp*). (c, d) During vernalization and de-acclimation conditions in hexaploid winter wheat seedlings (cv Norstar). After 2 weeks of germination at 20 ºC under LD conditions, non-vernalized winter wheat plants were vernalized under SD conditions at 4 ºC for 63 d and de-acclimated for 14 d at 20 ºC under LD conditions. The expression level of *TaFUL2* (panel c) and *TaFUL3* (panel d) are expressed relative to the non-vernalized plants (Ctrl-0). The aerial part was sampled around 4h after the beginning of the daylight period. (e, f) Effect of MeJA treatment in hexaploid spring wheat seedlings (Manitou). The expression level of *TaFUL2* (panel e) and *TaFUL3* (panel f) are expressed relative to the non-treated plants (Ctrl). Three weeks after germination at 20 ºC under LD conditions, control spring wheat (cv Manitou) plants (sprayed with 0.1% tween 20 solution only: Ctrl) were grown under LD conditions at 20 ºC for two weeks. Treated plants were sprayed with 150 μM MeJA dissolved in 0.1% tween solution every day for 2 weeks under the same growth conditions. Relative transcript abundance was calculated and normalized with respect to 18S *TaRNA* for the qRT-PCR experiment. Data represent the mean ± SEM from three biological replicates.

Previous results have shown that *FUL2* and *FUL3* are transcribed with an expression pattern similar to *VRN1* during vernalization (Chen and Dubcovsky, 2012). Thus, we verified if these two genes are present in our microarray data. The transcriptome of the *mvp* mutant indicates that *FUL3* has a high homology with the probeset TaAffx.120063.1.S1_at (Supplementary Table S2 available at *JXB* online) and corresponds to *TaAGL10*/*OsMADS18* (Zhao *et al.*, 2006). This gene was repressed 64-fold. We did not find a clear homologue for *FUL2* on the microarray. To verify whether these genes are present in the *mvp* mutant, we amplified *FUL2* and *FUL3* genes by PCR in *mvp*-mutant plants using genomic DNA. The data (not shown) indicate that both genes are not deleted in *mvp*-mutant plants. qRT-PCR analysis of these genes confirms that both genes (*FUL2* and *FUL3*) are highly downregulated in *mvp*-mutant plants compared with wild-type plants (Fig. 7a, b). In addition, their expression in winter wheat (cv. Norstar) is upregulated during vernalization and downregulated after de-acclimation under favourable growth conditions (Figs. 7c, d). This data is consistent with the results obtained by Chen and Dubcovsky (2012). More importantly, the expression of these two genes was repressed by MeJA (Fig. 7e, f).

Similarly, two other genes (*TaAGL33* and *TaAGL42*) within the MADS-box family were shown to be regulated by vernalization (Winfield *et al.*, 2009). In our transcriptome data, *TaAGL33* (corresponding to probeset Ta.26917.1.S1_at in Supplementary Table S2 available at *JXB* online) and *TaAGL42* (corresponding to probeset TaAffx.65068.1.A1_at in Supplementary Table S2 available at *JXB* online) were upregulated in *mvp*-mutant plants (3.08-fold and 4.6-fold respectively) compared with wild-type plants. Previous reports showed that the Jacalin-like lectin *VER2* is a vernalization-related gene that plays an important role in vernalization signalling and spike development in winter wheat and was considered as a jasmonate-regulated gene (Yong *et al.*, 2003). The microarray data indicate that two genes encoding putative jasmonate-induced protein are repressed –7.2-fold (corresponding to probesets Ta.7388.1.S1_at and Ta7388.2.S1._a_at; Table 1). However these two genes have no homology to *VER2*. A blast search of *VER2* (GenBank AB012103.3) against affymetrix database retrieved three genes with high homology: Ta.31.1.S1_at with a *P*-value of 1^e–149^, Ta.20205.2.S1_at with a *P*-value of 1^e–145^, and Ta.20205.1A1_s_at with a *P*-value of 1^e–123^. These three genes are not differentially regulated between wild-type and *mvp*-mutant plants.

**Fig. 8.**
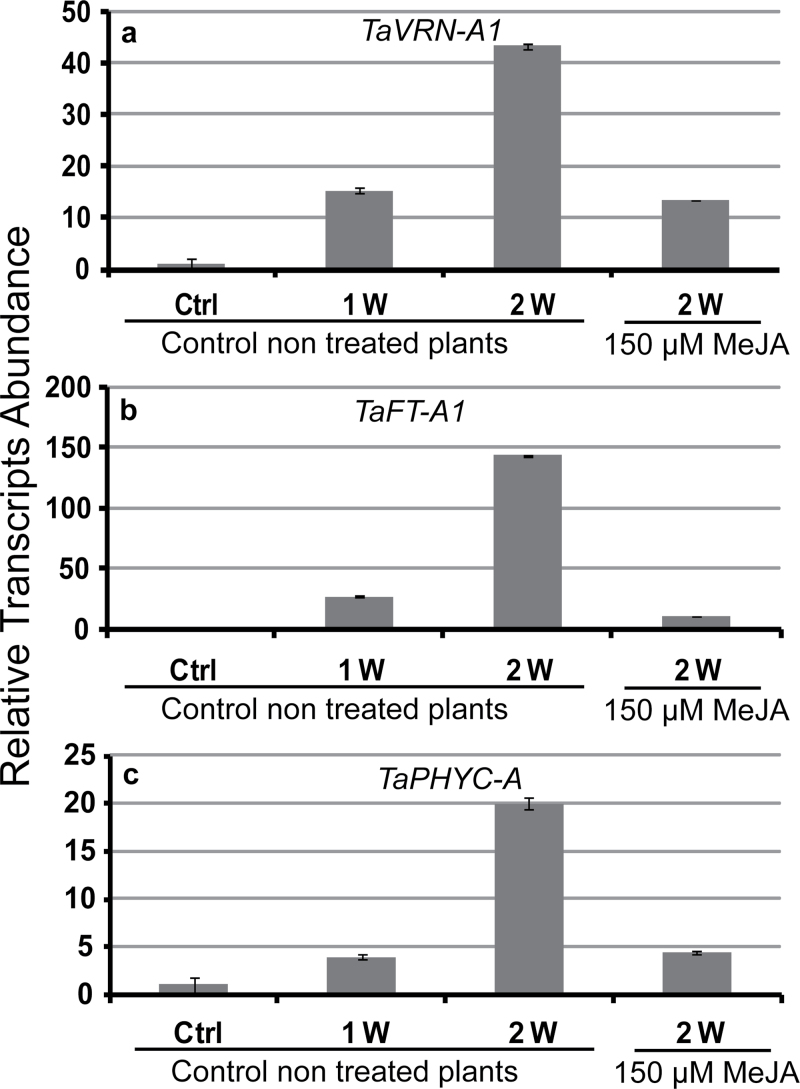
Effect of MeJA treatment on the expression of flowering-associated genes. Relative expression of flowering genes: (a) *TaVRN-A1*, (b) *TaFT-A1*, (c) *TaPHYC-A*. Three weeks after germination at 20 ºC under LD conditions, control spring wheat (cv Manitou) plants (treated with 0.1% tween 20 solution only: Ctrl) were grown under LD conditions at 20 ºC for two weeks. Treated plants were sprayed with 150 μM MeJA dissolved in 0.1% tween solution every day for 2 weeks under the same growth conditions. Total RNA was extracted from aerial parts and analysed by qRT-PCR. The 18S *TaRNA* was used as load control. Data represent the mean ± SEM from three biological replicates.

To test if exogenous MeJA caused flowering delay by acting on flowering genes, we measured the expression level of several wheat flowering genes in the vernalization-insensitive spring wheat cv Manitou (*TaVRN1*, *TaFT1*, and *TaPHYC*) before and at the end of MeJA (150 μM) treatment. The qRT-PCR results show that all flowering genes are repressed by MeJA compared with control non-treated plants which showed increased expression of all flowering genes in the first and second week of growth (Fig. 8). Furthermore, the repression of *TaFT1* by MeJA is more pronounced (Fig. 8b) indicating that this gene is more sensitive to MeJA than *TaVRN1* and *TaPHYC* (Fig. 8a, c). These results suggest that MeJA delays flowering by acting primarily through *TaFT1* repression. The expression profile of four biotic stress genes *TaLox2*, *TaFla*, *TaChi*, and *TaTha* known to be associated with jasmonate responses were measured using the same control and treated samples. Our results clearly show that the expression of the four genes is induced in treated plants compared with control plants (Supplementary Fig. S7, available at *JXB* online). Together our data indicate that accumulation of MeJA in plants is associated with flowering delay and shoot apical size reduction.

## Discussion

### The non-flowering *mvp* mutation elicits a biotic stress response and results in MeJA accumulation

The *mvp* mutant is incapable of going from the vegetative to the reproductive phase, owing to a deletion that includes the key vernalization gene *VRN1* (Shitsukawa *et al.*, 2007) and other genes (Distelfeld and Dubcovsky, 2010). The transcriptome analysis supports the finding that the *mvp* mutant has a deletion that includes the genes *CYS* and *PHYC* in addition to *VRN1* (Distelfeld and Dubcovsky, 2010), and another gene of unknown function that we named *UnG* (Fig. 3b). The mutation in *mvp* plants did not affect only flowering, but also other aspects of plant development such as root growth (Supplementary Fig. S1b, c, available at *JXB* online). In spite of this limitation, the *mvp* mutant remains a valuable tool to understand the function and regulation of *VRN1* genes in vernalization and flowering control.

Microarray analysis of cold-acclimated wild-type and *mvp*-mutant plants revealed that 368 genes are differentially regulated and are associated with several biochemical pathways. The 368 genes were classified into six major classes. Among the upregulated genes, several encode CBF transcription factors, cold acclimation induced protein, and an ice recrystallization inhibition protein. The upregulation of these genes and the accumulation of WCS120 and WCS19 proteins explain the enhanced freezing tolerance observed in *mvp* compared with wild-type plants (Supplementary Fig. S3, available at *JXB* online). These results are in agreement with a previous report showing that *mvp* plants have a higher freezing tolerance and increased transcript levels of several cold-regulated genes (Dhillon *et al.*, 2010). The higher freezing tolerance in the non-flowering *mvp* mutant supports the conclusion of Prášil *et al*. (2005) who suggested that genes involved in vernalization could influence the level and duration of freezing tolerance.

The most important molecular changes in the *mvp* mutant is the upregulation of genes that encode proteins related to biotic stress responses such as flavanone hydroxylase, pathogenesis-related protein PR-1a, chitinase, thaumatin, lipoxygenase, ice recrystallization inhibition protein, endochitinase, dehydrin, vacuolar defence protein, and defensin-like protein (Table 1). This change in expression of biotic stress genes is associated with the upregulation of *12-oxo-phytodienoic acid reductase* and *Lipoxygenase* suggesting the activation of jasmonate biosynthesis in *mvp*-mutant plants. This association suggests that upregulation of the biotic stress genes is elicited by the accumulation of jasmonates. Analyses of JA and MeJA content showed that MeJA content is six times higher in *mvp*-mutant compared with wild-type einkorn wheat confirming the activation of JA biosynthesis and suggesting that MeJA is the form that triggers the upregulation of biotic stress genes in the *mvp* mutant. MeJA is derived from JA and the reaction is catalysed by *S*-adenosyl-l-methionine:jasmonic acid carboxyl methyltransferase. This hormone has been identified as a vital cellular regulator that mediates diverse cellular responses, including defence, and developmental pathways, such as seed germination, root growth, flowering, fruit ripening, and senescence (Cheong and Choi, 2003). The inhibition of root growth was one of the first physiological effects detected for JA or its methyl ester form (Wasternack, 2007) and the accumulation of MeJA in the *mvp* mutant can thus explain the smaller root phenotype observed (Supplementary Fig. S1b, c, available at *JXB* online). The two forms of jasmonates are presumably interconvertible in plants by JA methyltransferase(s) (Seo *et al.*, 2001) and MeJA esterase(s) (Stuhlfelder *et al.*, 2004). However, the regulatory mechanism of MeJA biogenesis, and how it relates to jasmonate-responsive gene activation, is still unknown.

Jasmonates are known to activate plant defence mechanisms in response to biotic stress such as insect-driven wounding, various pathogens, and environmental stresses, such as drought, low temperature, and salinity by inducing the expression of WRKY transcription factors (Cheong and Choi, 2003; Houde *et al.*, 2006; Kim *et al.*, 2010; Li *et al.*, 2006). In *mvp* plants, several WRKY transcription factors were upregulated (Supplementary Table S2 available at *JXB* online) and some of these may be related to MeJA responses. Significant progress has been made on the characterization of WRKY proteins and many are involved in the regulation of plant defence responses (Eulgem and Somssich, 2007; Rushton *et al.*, 2010). Overexpression of *WRKY* genes in transgenic plants increased the production of PR proteins and disease resistance (Li *et al.*, 2006). WRKY transcription factors have broad roles in orchestrating metabolic responses to biotic stress, and they represent potentially valuable tools for engineering pathogen resistance (Naoumkina *et al.*, 2008). In addition to biotic stress genes, the sugar metabolism and oxidative stress genes are also modified in the *mvp* mutant. Soluble sugars, especially sucrose, glucose, and fructose, play a central role as nutrient and metabolite signalling molecules that activate specific or hormone crosstalk transduction pathways, thus resulting in important modifications of gene expression patterns in response to a number of stresses including pathogen attack (Couée *et al.*, 2006).

### MeJA is associated with vernalization and flowering time in wheat

Various physiological events in plants, such as defence responses, flowering, and senescence are mediated by jasmonates through intracellular and intercellular signalling pathways (Sasaki *et al.*, 2001). Jasmonate was shown to either promote or delay flowering depending on its concentration and on the plant species (Krajnčič *et al.*, 2006; Wasternack, 2007). The high concentration of MeJA in *mvp*-mutant plants suggested a possible role associated with flowering. To test this hypothesis, we measured the MeJA content in hexaploid winter wheat seedlings during vernalization to investigate whether MeJA is involved during the transition from vegetative to reproductive phases or during the final stages of flowering. Our results demonstrate that MeJA accumulates during vernalization in winter wheat and declines after vernalization completion when plants are grown under flowering inductive favourable conditions. At this last stage, *TaVRN1* reaches its maximal expression level, whereas MeJA is at its minimal level. The accumulation of MeJA begins early during vernalization before the accumulation of the major flowering gene *TaVRN1* and *TaFT1* (Fig. 4). During the cold period, MeJA may prevent the accumulation of *TaVRN1* and *TaFT1* from reaching its maximal level and thus delay flowering until favourable conditions for flowering are present (Fig. 4). The repression of *TaVRN1*, *TaFT1*, and the flowering delay in spring wheat was confirmed after exogenous application of MeJA. Taken together, these results suggest that the floral transition during or in response to vernalization is, at least in part, mediated by the accumulation of MeJA. The accumulation of MeJA during vernalization may also explain the induction of biotic stress genes (PR genes) needed to protect the plant during the low-temperature exposure required for vernalization (Houde *et al.*, 2006; Koike *et al.*, 2002). This conclusion is consistent with the optimal defence theory that predicts changes in the costs and benefits of the different types of defences over ontogeny (Diezel *et al.*, 2011).

The exact mechanism by which MeJA can delay flowering is still unknown and will need future investigation. Based on our finding and those of others, we can hypothesize that a MeJA-responsive transcription factor or complex may interact with the promoters of *FT1*, *VRN1*, and/or *PHYC* genes as the promoters of these three genes (*TaFT1*: gb_DQ890164.1; *TaVRN-A1*: gb_AY616452.1; *TaPHYC*: gb_AY672995.1) contain a *cis*-acting regulatory element involved in MeJA-responsiveness (CGTCA-motif and/or TGACG-motif) (Wang *et al.*, 2011). In *Arabidopsis*, it is reported that different genes are involved in both the flowering and the JA pathways. Overexpression of the HD-Zip II HAHB10 from *Helianthus annuus* in *Arabidopsis* led to early flowering and to a decreased level of JA after wounding compared with wild type. In transgenic plants overexpressing HAHB10, wounding led to the accumulation of JA and reduced the expression of HAHB10 (Dezar *et al.*, 2011). In *Arabidopsis*, Phytochrome and Flowering Time 1 (PFT1) also known as the MED25 subunit of the plant Mediator complex regulates flowering time downstream of the phytochrome phyB (Inigo *et al.*, 2012). PFT1 was recently proposed to act as a hub, integrating a variety of environmental stimuli including light quality and JA-dependent defences (Çevik *et al.*, 2012; Inigo *et al.*, 2012). Other subunits of the Mediator complex were also screened for their effect on plant defence and flowering time. The MED8 subunit was shown to have similar effects as MED25 indicating that more than one subunit of the Mediator complex is involved in the regulation of these two pathways (Kidd *et al.*, 2009). In both *med8* and *med25* mutants, the transcript level of *FT* was highly repressed, whereas the *FLC* transcript was highly expressed indicating that the major effect is on *FT* (Kidd *et al.*, 2009). These results are similar to the one found in wheat treated with MeJA, where the most downregulated gene was *TaFT1* (Fig. 8). The possible existence of MED25 (Ta.39294) homologues in wheat and the ability of the wheat MED25 (PFT1) to complement *med25* in *Arabidopsis* (Kidd *et al.*, 2009) suggest that MED25 may play a similar role in wheat and *Arabidopsis*. As a part of the Mediator complex, the MED25 subunit could be interacting with a relatively high number of transcription factors and mediate their effect on the transcription of target genes. MED25 was shown to interact with at least 10 transcription factors in the AP2-EREB, Myb, HD-ZF, and B-box families (Elfving *et al.*, 2011; Ou *et al.*, 2011). It was recently shown that MED25 is required for the transcription-activation ability of MYC2, ERF1, and ORA59, all known as important regulators of JA-associated pathogen- and herbivore-defence genes (Çevik *et al.*, 2012). The interaction of MED25 through the Mediator complex could thus provide a molecular mechanism by which JA could delay flowering in wheat treated with MeJA.

Overall our data provide evidence that the accumulation of MeJA during vernalization may play a dual role by stimulating biotic and abiotic stress defences to protect plants during winter and to delay flowering until growth conditions are favourable.

## Supplementary data

Supplementary data are available at *JXB* online

Table S1: Primers used for this study, with references or GenBank Accession Number.

Table S2: *mvp* wheat transcription factor differentially regulated genes identified by microarray.

Table S3: *mvp* wheat sugar-metabolism-related genes differentially regulated identified by microarray.

Table S4: *mvp* wheat oxidative-stress-related genes differentially regulated identified by microarray.

Table S5: *mvp* wheat miscellaneous genes differentially regulated identified by microarray.

Table S6: *mvp* wheat plant unknown genes differentially regulated identified by microarray.

Figure S1. Phenotype of maintained vegetative phase (*mvp*) plants and control plants (wild type).

Figure S2. Validation of microarray results with selected genes using qRT-PCR in *mvp* and wild-type control plants.

Figure S3. Expression level of COR proteins in wild-type and *mvp*-mutant plants before and after cold acclimation analysed by western blot.

Figure S4. Relative expression level of *TaLox2*, *TaFla*, *TaChi* and *TaTha* during vernalization and deacclimation conditions in hexaploid wheat seedlings analysed by qRT-PCR.

Figure S5. Effect of MeJA treatment on plant development in wild-type einkorn wheat.

Figure S6. Effect of MeJA treatment on plant development in *Triticum aestivum* wheat cv Manitou.

Figure S7. Effect of MeJA treatment on expression of PR genes *TaLox2*, *TaFla*, *TaChi* and *TaTha* analysed by qRT-PCR.

## Author contributions

AOD, ZA and FS conceived and designed the experiments. AOD, MH and FS wrote the manuscript. AOD identified the mvp mutant plants for both microarray experiment and the MeJA content measurement and prepared the figures. AOD and MH designed and analyzed the microarray data. AOD performed the experiments with ZA, MB and MAA-B. ZA conducted and performed the jasmonates treatments with MB, MAA-B and AOD. MAA-B performed the qPCR data analysis and AM performed the jasmonates content measurement. ZA, MB, MAA-B and AM critically read the manuscript.

## Supplementary Material

Supplementary Data
