# Supplementary material for: Transcriptome analysis of an mvp mutant reveals important changes in global gene expression and a role for methyl jasmonate in vernalization and flowering in wheat
Source: J Exp Bot. 2014 Mar 28;65(9):2271–86. doi: 10.1093/jxb/eru102 (PMC4036498; doi:10.1093/jxb/eru102)
Supplement: Supplementary Data [file supp_65_9_2271__index.html]

Transcriptome analysis of an mvp mutant reveals important changes in global gene expression and a role for methyl jasmonate in vernalization and flowering in wheat — Supplementary Data 

# Transcriptome analysis of an *mvp* mutant reveals important changes in global gene expression and a role for methyl jasmonate in vernalization and flowering in wheat

## Supplementary Data

Data files

**Files in this Data Supplement:**

- Supplementary Data - Supplementary Data
