# Supplementary material for: Transcriptome analysis of an mvp mutant reveals important changes in global gene expression and a role for methyl jasmonate in vernalization and flowering in wheat
Source: J Exp Bot. 2014 Mar 28;65(9):2271–86. doi: 10.1093/jxb/eru102 (PMC4036498; doi:10.1093/jxb/eru102)
Supplement: Supplementary Data [file supp_eru102_eru102_Supplementary_data.pdf]

Supporting information

Title: **Transcriptome analysis of *mvp* mutant reveals important changes in global gene expression and a role of methyl-jasmonate in vernalization and flowering in wheat**

Running title: **MeJA delays flowering in wheat**

Authors: Diallo, Amadou Oury*; Agharbaoui, Zahra*; Badawi, Mohamed A.; Ali-Benali, Mohamed Ali; Moheb, Amira; Houde, Mario and Sarhan, Fathey

* Equal contribution

Identification of mutant plants (*mvp*)

The homozygous *mvp* mutant plants were identified within the progeny of heterozygous parents by the absence of *VRN1* expression and confirmed by their inability to transit from vegetative to reproductive phases. For microarray analysis, the genotyping was performed as follows. First, potential *mvp* mutant plants (3 weeks old) were selected on the basis of their phenotype including plant size, internodes size, and number of emerged leaves. Potential *mvp* mutant and wild typeplants at the same age were acclimated for one week at 4°C under LD conditions. Then, the whole aerial part of each plant was individually sampled, quickly frozen and ground in dry ice and the powder was used for genomic DNA and total RNA extraction. Second, total RNA was isolated using TRIzol Reagent (Life Technologies) and analyzed by RT-PCR using specific primers of *VRN1*, *PHYC* and *CYSTEINE PROTEINASE* genes to confirm the absence of expression allowing us to select homozygous (*mvp / mvp*) or heterozygous (*Mvp / mvp*) mutant plants. Fifteen homozygous *mvp* mutant plants were randomly selected and used for molecular validation, microarray experiment and microarray results validation. Additional biological samples were also used for qRT-PCR and jasmonates quantification. After identification the homozygous *mvp* mutant plants, the same amount of total RNA from each *mvp* mutant sample was randomly pooled to produce 3 biological replicates, 5 *mvp* mutant plants per replicate. For WT plants, total RNA was extracted from 9 individual plants and the same amount of RNA was pooled to produce 3 biological replicates, 3 wild typeplants per replicate. For genomic DNA analysis, the same method was used to produce the 3 biological replicates using the powder from the same samples for each type of plants (*mvp* mutant and wild type plants) used in the microarray experiment. Genomic DNA from wild type and *mvp* mutant replicates were extracted to analyse a possible deletion of genes by PCR. Briefly, 0.1 g of powder for each replicate was

transferred in 1.5 mL eppendorf tube containing 500 µL of urea extraction buffer and 400 µL of phenol chloroform was added to the tube. After mixing, the tube was spun for 10 min at 10,000 *g*. The supernatant was transferred to a fresh tube and precipitated with 1 volume of isopropanol. The pellet was then dissolved by adding 500 µL Tris-EDTA (TE) buffer + 100 µL 4.4 M NH_4Acetate + 700 µL isopropanol. After mixing by inversion, the tube was spun at 10,000 *g* for 3 min. The DNA pellet was washed by adding 1 mL of ethanol 70%, spun again, dried and dissolved in 30 µL of TE buffer. DNA was then ready to be used for PCR analyses.

Freezing tolerance and COR proteins expression in *mvp* and wild type plants

Three-week-old soil-grown plants were cold acclimated at 4°C under the LD conditions. The non acclimated (NA) and cold acclimated (CA) plants were subjected to two separate freezing tests as described in details in our previous report (Diallo et al., 2010).

To test if the absence of *VRN1* and the other genes affect the ability of *mvp* plants to accumulate COR proteins, equal amounts of total proteins from the whole aerial part were used after extraction by grinding in a precooled motar with 0.1M TRIS-HCl, pH 8.5 containing 1 mM phenyl-methylsulfonyl fluoride (PMSF). The extract was centrifuged for 5 min at 14,000 *g* at 4°C and the supernatant was adjusted to 1X final buffer concentration using 2X SDS electrophoresis sample buffer (Laemmli, 1970). Samples were separated on a 12% SDS-polyacrylamide gel and transferred electrophoretically for 1 hour to a 0.45 µm polyvinylidene fluoride (PVDF) membrane (Life Sciences BioTrace™). The membrane was blocked in a 4% (w/v) solution of reconstituted skimmed milk powder prepared in PBS containing 0.2% (v/v) Tween 20 and then probed with different antibodies: anti-Wheat cold specific 120 (WCS120) at 1: 20,000; anti-Wheat cold specific 19 (WCS19) at 1: 10,000; and anti-Wheat cold-regulated 410 (WCOR 410) at 1: 5,000 dilution overnight. After washing three times 20 min with PBS–Tween, the proteins recognized by the primary antibody were

revealed with a goat anti-rabbit HRP conjugated IgG (EMD Millipore, Catalog # 12-348) at a dilution of 1: 20,000. The complexes were visualized using the HyGLO™ Chemiluminescent HRP Antibody Detection Reagent and HyBlot CL™ Autoradiography Film (Cat. No. E3018 DENVILLE Scientific Inc. P.O. Box 4588 Metuchen, NJ 08840-4588 USA).

Quantification of jasmonates

500 mg from powdered plants were extracted twice using 2 mL of MeOH/H_20/HOAC (90:9:1 v/v/v) (Segarra et al., 2006). The tubes were shaken on a vortex for 2 h at 4ºC, and centrifuged at 1500*g* for 5 min at 4ºC. The supernatant was dried under a gentle stream of N_2 and the dried powder reextracted by adding 250 µl of MeOH and then 250 µl of MeOH/H_2O (15:85). Samples were filtered on 0.4 µm syringe mini filters before being injected into HPLC/MS. Benzoic acid was used as internal standard (Hou et al., 2008). LC-MS analyses were carried using an Agilent 1200 HPLC system with binary pump, in-line degasser, high performance auto-sampler and thermostated column division, using a linear gradient of 20% to 95% methanol: 0.1% HCOOH /water for 20 min with a flow rate of 0.35 ml/min on an Agilent SB-C18 column (2.1 ×30mm; particle size, 3.5µm) and a column temperature of 25ºC. The HPLC instrument was connected to an Agilent 6410 quadrupole (triple Q) mass spectrometer using electrospray ionisation in both negative and positive ESI mode for the analysis of jasmonic acid and its methyl ester, respectively, with the following conditions: capillary voltage: 3000 volts, nebulizer pressure: 60 PSI, gas temperature 300ºC, drying gas: 5 L/min and a dwell time of 75 ms, and the data was processed using the Mass Hunter software. For MS/MS and MRM analyses, the collision energies (CE) were optimized; a (CE) of - 5eV and + 25 eV was applied as the optimum energy of collision for the analysis of jasmonic acid and methyl jasmonate, respectively. The scan range was from 200 to 900 m/z. For jasmonic acid (JA), MRM acquisition was carried out by monitoring transitions of the

combination of the parent ion mass in negative mode, 209 m/z and the fragment ion of highest abundance 59; whereas the transition was monitored in positive mode between 225 and 133 for methyl jasmonate (MeJA). References of JA and MeJA were used to compare the corresponding retention times and mass spectral profiles. An external calibration curve was constructed using different concentrations of JA and MeJA for quantification purposes, along with the use of benzoic acid as internal standard. The data presented is the average +/- SE of three biological replicates. The experiment was repeated at least two times (new biological replicates) with three technical replicates per experiment.

Table S1: **Primers used for this study, with references or GenBank Accession Number**

**Primers GenBank Accession Number or Reference**
**RT-PCR study**
5’-AATATACACGCGCCACATCA-3’*TmCir-FW***AK334473**
5’-CAAGATGTCACAGCAGCACA-3 *TmCir-RV*
5’-AGGCCTTCTGACAACTGGAA-3’*TmUnG-FW***CA646083**
5’-TGTGTGCCACAGCAGTACAA-3’*TmUnG-RV*
5’-GGAGCAGAGGCAACTTTTTG-3’*TmPHYC-FW***AY244514**
5’-ATTCCACCGTGTTCATCTCC-3’ *TmPHYC-RV*
5’-TCGTGGAGAAGCAGAAGGC-3’ *VRN1-BAC 81655L* **Shimada et al 2009**
5’-GTTGATGTGGCTCACCATCC-3’ *VRN1-BAC 82017R*
5’-TAAGAAGGAAGGGGGAATGG-3’ *WFT-FW***Shimada et al 2009**
5’-GAGGGCTCTCGTAGCACATC-3’ *WFT-RV*
**RT-PCR and qRT-PCR studies**
5’-ATGCCTAGTAAGCGCGAGTCAT-3’*18S_RNA-FW***Diallo et al 2010**
5’-ACGGGCGGTGTGTACAAAG-3’*18S_RNA-RV*
**qRT-PCR study**
5’-CAGGCCGGTCGATCTATACTA-3’*WFT-F4***Shimada et al 2009**
5’-TCCTGTTCCCGAAGGTCA-3’*WFT-R4*
5’-GGAGAGGTCACTGCAGGAGGA-3’*WAP1-545L***Shimada et al 2009**
5’-GCCGCTGGATGAATGCTG-3’*WAP1-698R*
5’-GAAGGTCAGAAGATGTGGAGAGTCAAC-3’ *TaGI-3L***Shimada et al 2009**
5’-GGCAGCGGATGGTAGGTGATAG-3’ *TaGI-3R*
5’-ATGTCACCCCAGGTTATTGG-3’*qTmCYS-F***AY244511**
5’-TTTTCGGTCAACCCAGACAG-3’ *qTmCYS-R*
5’-ATCCACGACGCAGCATTAC-3’*qTaFlav-F***DQ208192**
5’-GTTGTGCTCAAGCAAAAACAAG-3’ *qTaFlav-R*
5’-GCCCATCTGGTGTGTGTTC-3’,*qTaOMT-F***BT009571**
5’-CCATTGACGAGTAAGGCACTG-3’, *qTaOMT-R*
5’-TGTGTCTGTGCTGATGCAGA-3’*qTmPHYC-F***AY244514**
5’-GGCATGCTAAACTGTTGTGTG-3’*qTmPHYC-R*
5’-CCCTCACTGTTTATTTCCCTGTG-3’*qTmCir-F***AK334473**
5’-GAATGAGACGGCAGACACT-3’ *qTmCir-R*
5’-CCCAACAGCATCTCCATTTG-3’*qTdLOX-B1-F***AK333416**
5’-TAGTTCAGCCACACACACACAC-3’*qTdLOX-B1-R*
5’-CCATACAAAAATGTCACAAGC-3’ q*TaFUL*2-F **Chen and Dubcovsky, 2012**
5’-TTCTGCCTCTCCACCAGTTC-3’ *qTaFUL2-R*
5’-ATGGATGTGATTCTTGAACG-3’ *qTaFLU3-F*
5’-AGTTGCCTTTGACTCTTCTG-3’ *qTaFUL3-R*

Table S2*: mvp* wheat transcription factor differentially regulated genes identified by microarray

Affymetrix probeset IDs	GenBank Accession	UniProt and NCBI Description	Fold Change
Ta.26917.1.S1_at	CD452828	MADS-box transcription factor TaAGL41 n=2 Tax=Triticum aestivum RepID=Q1G168_WHEAT	4.600
TaAffx.122374.1.A1_at	BE517594	CBFIVa-2.2 (Fragment) n=1 Tax=Triticum aestivum RepID=A0MPK8_WHEAT	3.525
TaAffx.109191.1.S1_at	BQ168959	WRKY transcription factor n=1 Tax=Triticum aestivum RepID=A3FBG2_WHEAT	3.146
TaAffx.65068.1.A1_at	BJ263737	MADS-box transcription factor TaAGL42 n=1 Tax=Triticum aestivum RepID=Q1G167_WHEAT	3.081
TaAffx.109191.1.S1_x_at	BQ168959	WRKY transcription factor n=1 Tax=Triticum aestivum RepID=A3FBG2_WHEAT	2.808
TaAffx.98930.1.A1_at	CK214676	CBF12 n=8 Tax=Triticeae RepID=B1NSN2_TRIMO	2.242
Ta.8614.1.S1_at	BQ838257	WRKY45 transcription factor n=1 Tax=Triticum aestivum RepID=A3RG93_WHEAT	2.237
Ta.4725.1.S1_at	CK195830	WRKY14 transcription factor n=1 Tax=Triticum aestivum RepID=B3GAU5_WHEAT	2.207
Ta.30495.1.A1_s_at	CK214893	CBFIVc-14.1 n=2 Tax=Triticum RepID=A0MPL5_WHEAT	2.176
Ta.21250.1.S1_at	BJ273029	MADS-box transcription factor TaAGL23 n=4 Tax=Triticeae RepID=Q1G185_WHEAT	2.144
TaAffx.80313.1.S1_at	CA698434	Putative WRKY5 protein (Fragment) n=1 Tax=Hordeum vulgare RepID=Q5W1F7_HORVU	2.094
Ta.6374.3.S1_a_at	BJ300310	Squamosa promoter-binding-like protein 13 n=3 Tax=Oryza sativa RepID=SPL13_ORYSJ	-2.092
TaAffx.143995.17.A1_at	AY188331	MADS box transcription factor n=15 Tax=Triticeae RepID=O82128_WHEAT	-2.714
Ta.9216.1.A1_a_at	BQ165936	Homeobox-leucine zipper protein HOX12 n=3 Tax=Oryza sativa RepID=HOX12_ORYSJ	-2.813
Ta.30640.1.S1_at	CD861747	Triticum aestivum flowering locus T mRNA, complete cds or VRN3	-3.250
Ta.246.1.S1_at	AF224499	Homeobox protein KNOX3 n=3 Tax=Triticeae RepID=KNOX3_HORVU	-3.477
Ta.9216.1.A1_x_at	BQ165936	Homeobox-leucine zipper protein HOX12 n=3 Tax=Oryza sativa RepID=HOX12_ORYSJ	-3.497
Ta.3583.1.A1_at	BJ219585	MADS-box protein 8 n=4 Tax=Pooideae RepID=Q9LEI0_HORVU	-5.963
TaAffx.120063.2.S1_s_at	BJ245749	MADS-box transcription factor 18 n=4 Tax=Oryza sativa RepID=MAD18_ORYSJ	-37.146
Ta.30607.1.A1_at	BJ264278	MADS box transcription factor n=15 Tax=Triticeae RepID=O82128_WHEAT	-60.556
TaAffx.120063.1.S1_at	CF134093	MADS-box transcription factor 18 n=4 Tax=Oryza sativa RepID=MAD18_ORYSJ	-64.352
Ta.6793.1.A1_at	CD492136	MADS2 n=3 Tax=Triticeae RepID=A5X498_WHEAT	-105.103
TaAffx.143995.17.S1_s_at	AY188331	MADS box transcription factor n=15 Tax=Triticeae RepID=O82128_WHEAT	-108.744

Legend: The annotation is made according to Affymetrix Gene Chip® wheat genome array of the 23 Transcription factors probesetIDs complemented with BLAST results showing the Genbank accession number, UniProt or NCBI description and is presented in decreasing order of differential expression of ≥ 2 -fold and $\leq$ -2-fold cut off.

Table S3:*mvp* wheat sugar metabolism related genes differentially regulated identified by microarray

Affymetrix probeset IDs	GenBank Accession	UniProt and NCBI Description	Fold Change
TaAffx.107485.1.S1_at	CA699183	Glucomannan 4-beta-mannosyltransferase 1 n=3 Tax=Oryza sativa RepID=CSLA1_ORYSJ	6.833
Ta.26048.1.S1_x_at	CD454944	Beta-glucanase n=1 Tax=Hordeum vulgare RepID=Q7M1K2_HORVU	6.647
TaAffx.15327.1.S1_at	AJ610775	Glucan endo-1,3-beta-glucosidase GII n=9 Tax=Triticeae RepID=E13B_HORVU	3.959
Ta.22565.1.S1_at	BT009372	UDP-glucosyl transferase n=1 Tax=Triticum aestivum RepID=C5HUX8_WHEAT	3.453
TaAffx.24475.1.S1_at	CA718969	Glucan endo-1,3-beta-glucosidase GII n=9 Tax=Triticeae RepID=E13B_HORVU	3.382
Ta.22565.1.S1_x_at	BT009372	UDP-glucosyl transferase n=1 Tax=Triticum aestivum RepID=C5HUX8_WHEAT	3.141
TaAffx.24475.1.S1_x_at	CA718969	Glucan endo-1,3-beta-glucosidase GII n=9 Tax=Triticeae RepID=E13B_HORVU	3.123
Ta.12517.1.S1_at	CK163074	Sugar transport protein 1 n=2 Tax=Zea mays RepID=Q6B4G9_MAIZE	2.900
Ta.3828.3.A1_x_at	BJ254343	Putative glucan endo-1,3-beta-D-glucosidase (Fragment) n=1 Tax=Triticum aestivum	2.596
Ta.223.1.S1_at	AF112965	Beta-1,3-glucanase n=1 Tax=Triticum aestivum RepID=Q9XEN5_WHEAT	2.464
TaAffx.20635.1.S1_at	CD895869	Sugar transporter family protein, expressed n=3 Tax=Oryza sativa RepID=Q10QG4_ORYSJ	2.319
Ta.3828.3.A1_a_at	BJ254343	Putative glucan endo-1,3-beta-D-glucosidase (Fragment) n=1 Tax=Triticum aestivum	2.018
TaAffx.15847.3.S1_at	BQ802374	Xyloglucan endotransglycosylase (XET) n=1 Tax=Hordeum vulgare RepID=P93671_HORVU	-2.200
Ta.9000.1.S1_at	CD453515	Acid beta-fructofuranosidase n=1 Tax=Triticum aestivum RepID=Q575T1_WHEAT	-2.378
Ta.9058.1.S1_at	CA642666	Acid beta-fructofuranosidase n=1 Tax=Triticum aestivum RepID=Q575T1_WHEAT	-3.184
TaAffx.410.1.S1_s_at	CA646394	Sucrose synthase metabolism (Fragment) n=3 Tax=Poaceae RepID=A6MZV1_ORYSI	-3.263
TaAffx.410.1.S1_at	CA646394	Sucrose synthase metabolism (Fragment) n=3 Tax=Poaceae RepID=A6MZV1_ORYSI	-3.519

Legend: The annotation is made according to Affymetrix Gene Chip® wheat genome array of the 17 sugar metabolism related probesetIDs differentially regulated complemented with BLAST results showing the Genbank accession number, UniProt or NCBI description and is presented in decreasing order of differential expression of $\geq$ 2-fold and $\leq$ -2-fold cut off.

Table S4:*mvp* wheat oxidative stress related genes differentially regulated identified by microarray

Affymetrix probeset IDs	GenBank Accession	UniProt and NCBI Description	Fold Change
TaAffx.1074.1.S1_at	CK212638	Cytochrome c oxidase subunit 1 n=9 Tax=Aphidomorpha RepID=Q69HZ8_9HEMI	3.884
TaAffx.100029.1.S1_at	CA678048	Leucoanthocyanidin dioxygenase n=1 Tax=Zea mays RepID=B6U9L0_MAIZE	3.377
Ta.5235.1.S1_x_at	BG606752	Peroxidase n=2 Tax=Triticeae RepID=O49866_HORVU	3.056
Ta.5385.1.S1_at	X85228	Peroxidase 1 n=8 Tax=Triticeae RepID=PER1_HORVU	2.933
TaAffx.50125.2.S1_at	CA745672	Cytochrome P450 n=1 Tax=Triticum aestivum RepID=Q9AVM3_WHEAT	2.707
TaAffx.85775.1.S1_at	CA621227	Cytochrome P450 monooxygenase CYP71U4v2 n=2 Tax=Hordeum vulgare RepID=Q52PG2_HORVD	2.680
Ta.24106.1.S1_x_at	CA665159	Peroxidase 1 n=8 Tax=Triticeae RepID=PER1_HORVU	2.569
TaAffx.115935.1.S1_x_at	AY596267	Polyphenol oxidase n=2 Tax=Triticum aestivum RepID=C0SPI5_WHEAT	2.515
TaAffx.65294.1.A1_at	BJ252171	Chloroplast lipocalin n=2 Tax=Triticeae RepID=Q38JB3_WHEAT	2.437
Ta.3703.2.S1_s_at	BJ246711	Cytochrome P450 CYP709E4 n=2 Tax=Zea mays RepID=B6TF97_MAIZE	2.431
Ta.20570.1.A1_at	CA741536	1-aminocyclopropane-1-carboxylate oxidase (Fragment) n=2 Tax=Andropogoneae RepID=O81607_SORBI	2.411
Ta.23366.3.A1_x_at	BQ161967	Class III peroxidase 62 n=2 Tax=Oryza sativa Japonica Group RepID=Q5U1N1_ORYSJ	2.400
TaAffx.80306.1.S1_at	CA698528	1-aminocyclopropane-1-carboxylate oxidase (Fragment) n=1 Tax=Triticum monococcum	2.322
Ta.23366.3.A1_at	BQ161967	Class III peroxidase 62 n=2 Tax=Oryza sativa Japonica Group RepID=Q5U1N1_ORYSJ	2.290
Ta.29496.1.S1_x_at	CK195383	Peroxidase 12 n=2 Tax=Zea mays RepID=B6THG0_MAIZE	2.281
Ta.21505.1.S1_at	CK213957	Root peroxidase n=1 Tax=Triticum aestivum RepID=B4F6E8_WHEAT	2.276
Ta.21307.1.S1_x_at	CK199589	Peroxidase 12 n=2 Tax=Zea mays RepID=B6THG0_MAIZE	2.186
Ta.303.3.S1_x_at	AJ610447	Glutathione S-transferase 2 n=2 Tax=Triticum aestivum RepID=GSTF2_WHEAT	2.175
Ta.24710.1.S1_at	CA678411	Peroxidase 1 n=8 Tax=Triticeae RepID=PER1_HORVU	2.087
Ta.10549.2.A1_at	BQ165963	Alternative oxidase n=3 Tax=Oryza sativa RepID=O82807_ORYSJ	2.050
Ta.19609.1.S1_at	AJ615628	Cytochrome P450 n=1 Tax=Triticum aestivum RepID=Q2V065_WHEAT	2.015

Legend: The annotation is made according to Affymetrix Gene Chip® wheat genome array of the 21 oxidative stress related probesetIDs up-regulated complemented with BLAST results showing the Genbank accession number, UniProt or NCBI description and is presented in decreasing order of differential expression of ≥ 2-fold and $\leq$ -2-fold cut off.

Table S5:*mvp* wheat miscellaneous genes differentially regulated identified by microarray

Affymetrix probeset IDs	GenBank Accession	UniProt and NCBI Description	Fold Change
TaAffx.57571.1.S1_at	CA626944	Apyrase n=1 Tax=Lolium perenne RepID=B9U140_LOLPR	31.363
TaAffx.70677.1.S1_at	CA701525	Chaperone protein dnaJ n=2 Tax=Andropogoneae RepID=B4FBY1_MAIZE	7.753
TaAffx.89610.1.S1_at	AL823263	Agmatine coumaroyltransferase n=1 Tax=Zea mays RepID=B6ST92_MAIZE	6.540
Ta.27314.1.S1_at	BT009398	BRASSINOSTEROID INSENSITIVE 1-associated receptor kinase 1 n=1 Tax=Zea mays	6.180
Ta.8902.2.S1_at	CA640147	Taxane 13-alpha-hydroxylase n=1 Tax=Zea mays RepID=B6SYR0_MAIZE	5.532
Ta.28917.2.S1_a_at	CA637102	Cold acclimation protein WCOR518 (Fragment) n=1 Tax=Triticum aestivum RepID=P93611_WHEAT	4.341
Ta.25531.1.A1_at	CD373817	Serine/threonine kinase-like protein n=3 Tax=Andropogoneae RepID=B6SUE5_MAIZE	4.302
Ta.18720.1.S1_x_at	BJ288588	Gamma-thionin n=1 Tax=Hordeum vulgare RepID=Q39999_HORVU	3.753
Ta.8902.1.S1_at	BQ162604	Taxane 13-alpha-hydroxylase n=1 Tax=Zea mays RepID=B6SYR0_MAIZE	3.703
TaAffx.132143.1.S1_s_at	CK212211	Cyanate hydratase n=3 Tax=Oryza sativa RepID=CYNS_ORYSJ	3.432
Ta.25531.2.A1_x_at	CA680295	Serine/threonine kinase-like protein n=3 Tax=Andropogoneae RepID=B6SUE5_MAIZE	3.426
Ta.9220.1.S1_a_at	CK210556	Putative phenylalanine ammonia-lyase (Fragment) n=1 Tax=Hordeum vulgare	3.352
TaAffx.70601.1.S1_at	BQ802878	Triticum aestivum cultivar 92R137 PDR-type ABC transporter (PDR1)	3.344
Ta.8232.1.A1_at	BQ161629	PDR-type ABC transporter n=2 Tax=Triticum aestivum RepID=B9UYP3_WHEAT	3.309
TaAffx.97211.1.S1_at	AJ614654	O-methyltransferase n=1 Tax=Triticum aestivum RepID=B4ERX7_WHEAT	3.268
Ta.13784.1.S1_at	AL823038	BLT14.1 protein n=2 Tax=Triticeae RepID=Q40032_HORVU	3.178
TaAffx.110222.1.S1_x_at	CA661932	BRASSINOSTEROID INSENSITIVE 1-associated receptor kinase 1 n=1 Tax=Zea mays RepID=B6STG1_MAIZE	3.144
Ta.28917.1.S1_at	CK166116	Cold acclimation protein WCOR518 (Fragment) n=1 Tax=Triticum aestivum RepID=P93611_WHEAT	3.099
Ta.21646.1.S1_x_at	CA686121	Non-specific lipid-transfer protein n=3 Tax=Oryza sativa RepID=Q75GN2_ORYSJ	3.071
Ta.28917.1.S1_x_at	CK166116	Cold acclimation protein WCOR518 (Fragment) n=1 Tax=Triticum aestivum RepID=P93611_WHEAT	2.978
TaAffx.113315.1.S1_at	CA615901	Iron-phytosiderophore transporter n=1 Tax=Hordeum vulgare RepID=Q2PGC4_HORVU	2.967
Ta.25531.2.A1_at	CA680295	Serine/threonine kinase-like protein n=3 Tax=Andropogoneae RepID=B6SUE5_MAIZE	2.964
Ta.18203.1.S1_at	AF031195	Blue copper-binding protein homolog n=1 Tax=Triticum aestivum RepID=Q9ZTU7_WHEAT	2.824
Ta.24730.2.S1_x_at	BE430562	Protochlorophyllide reductase (Fragment) n=4 Tax=Poaceae RepID=POR_AVESA	2.743
TaAffx.16032.1.A1_at	BQ800652	Cell wall invertase n=1 Tax=Lolium perenne RepID=Q2QI10_LOLPR	2.730
Ta.28613.1.S1_at	U73213	Cold acclimation protein WCOR726 n=3 Tax=Triticeae RepID=P93610_WHEAT	2.708
Ta.5654.1.S1_at	BJ286960	Blue copper-binding protein n=1 Tax=Dasypyrum villosum RepID=A7UHA3_9POAL	2.637
TaAffx.120890.1.S1_x_at	CA688266	Serine/threonine kinase-like protein n=3 Tax=Andropogoneae RepID=B6SUE5_MAIZE	2.575
Ta.11016.1.S1_at	BQ168385	Calmodulin-like protein n=1 Tax=Zea mays RepID=B6TXW9_MAIZE	2.565
TaAffx.55592.1.S1_at	CA663917	Potassium transporter 5 n=1 Tax=Oryza sativa Japonica Group RepID=HAK5_ORYSJ	2.481
Ta.21272.1.S1_at	CA668970	Cell division AAA ATPase family protein n=10 Tax=Triticeae RepID=B6Z264_WHEAT	2.470
Ta.8228.1.S1_at	BQ161624	Agmatine coumaroyltransferase n=3 Tax=Hordeum vulgare RepID=A9ZPJ6_HORVU	2.432
Ta.351.2.S1_x_at	CA673444	Cold acclimation induced protein 2-1 n=1 Tax=Triticum aestivum RepID=Q4KXE0_WHEAT	2.400
Ta.23397.1.S1_x_at	BJ292777	Glycine-rich cell wall structural protein n=1 Tax=Hordeum vulgare RepID=GRP1_HORVU	2.390
Ta.23397.2.S1_x_at	CA718270	Pherophorin-dz1 protein n=1 Tax=Volvox carteri f. nagariensis RepID=Q8L685_VOLCA	2.384
Ta.28659.1.S1_x_at	CA670381	Putative protease inhibitor n=1 Tax=Hordeum vulgare RepID=Q96465_HORVU	2.384
Ta.18720.3.S1_x_at	BJ295779	Gamma-thionin n=1 Tax=Hordeum vulgare RepID=Q39999_HORVU	2.357
Ta.21650.1.A1_at	CA667760	NBS-LRR resistance-like protein n=1 Tax=Hordeum vulgare RepID=A9UKM2_HORVU	2.343
Ta.24254.1.S1_a_at	CA616263	Plastid omega-3 fatty acid desaturase n=2 Tax=Oryza sativa Japonica Group RepID=Q2HWS9_ORYSJ	2.319
Ta.18720.1.S1_a_at	BJ288588	Gamma-thionin n=1 Tax=Hordeum vulgare RepID=Q39999_HORVU	2.267

Ta.8512.1.S1_at	BQ162001	Os07g0631700 protein n=2 Tax=Oryza sativa RepID=Q8LHN5_ORYSJ	2.254
Ta.6187.1.S1_at	BQ167028	Glycine-rich cell wall structural protein n=2 Tax=Zea mays RepID=B6ST85_MAIZE	2.249
Ta.351.1.S1_at	CA669389	Cold acclimation induced protein 2-1 n=1 Tax=Triticum aestivum RepID=Q4KXE0_WHEAT	2.247
TaAffx.113701.1.S1_s_at	CA606782	Pleiotropic drug resistance protein 4 n=3 Tax=Oryza sativa RepID=PDR4_ORYSJ	2.172
Ta.169.1.S1_x_at	CA652856	Germin-like 12 n=7 Tax=Triticeae RepID=Q43487_HORVU	2.170
TaAffx.3462.1.S1_at	CK215505	Early salt stress and cold acclimation-induced protein 2-1 n=1 Tax=Lophopyrum elongatum	2.161
Ta.27506.1.S1_at	AY428038	Ammonium transporter AMT2.1 n=1 Tax=Triticum aestivum RepID=Q6T8L6_WHEAT	2.155
Ta.2793.1.S1_at	AB055077	Multidrug resistance protein 1 homolog n=1 Tax=Triticum aestivum RepID=Q8RVT7_WHEAT	2.151
TaAffx.77715.1.S1_at	CA744342	S-domain receptor-like protein kinase n=1 Tax=Oryza granulata RepID=B9V0L1_9ORYZ	2.151
Ta.6990.1.S1_at	CA611113	Pleiotropic drug resistance protein 4 n=3 Tax=Oryza sativa RepID=PDR4_ORYSJ	2.133
TaAffx.81369.1.S1_at	CA686927	Receptor-like protein kinase n=2 Tax=Oryza sativa RepID=Q9M575_ORYSA	2.099
Ta.27258.1.S1_at	BT009386	BRASSINOSTEROID INSENSITIVE 1-associated receptor kinase 1 n=1 Tax=Zea mays	2.091
Ta.14837.1.S1_at	CA682925	Calreticulin, putative n=1 Tax=Ricinus communis RepID=B9RM48_RICCO	2.075
Ta.3154.1.S1_at	BJ223797	Sorghum bicolor hypothetical protein (SORBIDRAFT_0019s004610) mRNA	2.043
Ta.24723.1.S1_x_at	CA667447	PSBGer1 protein n=1 Tax=Triticum aestivum RepID=P93598_WHEAT	2.043
TaAffx.56501.1.S1_at	BU099852	Sorghum bicolor hypothetical protein (SORBIDRAFT_0019s004610) mRNA, complete cds	2.026
TaAffx.128683.1.S1_x_at	CA684810	Cell division AAA ATPase family protein n=10 Tax=Triticeae RepID=B6Z264_WHEAT	2.022
Ta.22101.1.A1_at	CA721955	Chlorophyll a-b binding protein 22L, chloroplastic n=14 Tax=Solanaceae	-2.022
Ta.26236.1.A1_at	CD453043	Abhydrolase domain-containing protein 5 n=2 Tax=Andropogoneae RepID=B4FAQ7_MAIZE	2.005
Ta.28827.1.S1_at	BQ800700	Histone H1 n=2 Tax=Triticum aestivum RepID=O65795_WHEAT	2.004
Ta.1282.4.S1_at	CA635994	Non-specific lipid-transfer protein n=3 Tax=Triticeae RepID=Q42848_HORVU	-2.017
TaAffx.95414.1.S1_at	BQ803340	Branched-chain-amino-acid aminotransferase n=1 Tax=Hordeum vulgare	-2.020
Ta.2969.1.A1_at	CK165153	Expansin EXPB2 n=1 Tax=Triticum aestivum RepID=Q6QFA2_WHEAT	-2.024
Ta.28728.1.S1_at	AF139815	Aquaporin PIP2-4 n=9 Tax=Poaceae RepID=PIP24_MAIZE	-2.035
TaAffx.113526.2.S1_s_at	CA723504	Proline-rich protein n=2 Tax=Triticum aestivum RepID=C7EZF0_WHEAT	-2.081
Ta.28728.1.S1_x_at	AF139815	Aquaporin PIP2-4 n=9 Tax=Poaceae RepID=PIP24_MAIZE	-2.093
TaAffx.8804.2.S1_at	BE217049	Probable aquaporin PIP2-7 n=3 Tax=Oryza sativa RepID=PIP27_ORYSJ	-2.144
Ta.30144.1.A1_x_at	BQ166180	Proline-rich protein n=1 Tax=Sorghum bicolor RepID=A7KH48_SORBI	-2.190
Ta.3.1.S1_at	BJ265463	Beta-amylase n=2 Tax=Triticeae RepID=AMYB_WHEAT	-2.226
TaAffx.43693.1.S1_at	BF199968	Xet3 protein n=1 Tax=Festuca pratensis RepID=Q949H9_FESPR	-2.268
TaAffx.71208.1.S1_at	BE492167	Amidase n=2 Tax=Zea mays RepID=B6TMI1_MAIZE	-2.269
TaAffx.8804.1.S1_s_at	CK164001	Probable aquaporin PIP2-7 n=3 Tax=Oryza sativa RepID=PIP27_ORYSJ	-2.287
Ta.13160.1.S1_at	CK195065	Stem-specific protein n=4 Tax=Andropogoneae RepID=Q5U7K5_9POAL	-2.289
Ta.1600.1.A1_at	CK164799	Nodulin-like protein 5NG4 n=3 Tax=Andropogoneae RepID=B6TLA9_MAIZE	-2.301
Ta.10400.1.S1_at	BJ251360	Cortical cell-delineating protein n=7 Tax=Zea mays RepID=B6SIN6_MAIZE	-2.324
TaAffx.632.1.A1_at	CD490405	Hordeum vulgare ENOD40-like protein mRNA, complete cds	-2.325
TaAffx.36760.1.S1_at	BJ315664	CEN-like protein 2, putative, expressed n=13 Tax=Poaceae RepID=Q53Q71_ORYSJ	-2.392
Ta.346.1.A1_at	BJ254518	Cycloartenol synthase, putative, expressed n=1 Tax=Oryza sativa Japonica Group	-2.616
TaAffx.16936.1.S1_at	CK216481	Fasciclin-like protein FLA16 n=2 Tax=Triticum aestivum RepID=Q06I90_WHEAT	-2.665
Ta.20696.3.S1_x_at	CA598744	Circumsporozoite protein n=2 Tax=Andropogoneae RepID=B6TF33_MAIZE	-2.767
Ta.438.2.S1_x_at	CK212622	Nuclease PA3 n=3 Tax=Zea mays RepID=B6U2F0_MAIZE	-3.276
Ta.22954.1.S1_at	CA673824	Putative thionin Osthi1 n=2 Tax=Oryza sativa RepID=Q5Z4W6_ORYSJ	-3.384
Ta.438.1.S1_x_at	BG908651	Nuclease PA3 n=3 Tax=Zea mays RepID=B6U2F0_MAIZE	-3.451
Ta.22954.2.S1_x_at	CA675346	gene="ACT-1" actin [Triticum aestivum] protein_id="AAW78915.1	-3.497

Ta.438.1.S1_a_at	BG908651	Nuclease PA3 n=3 Tax=Zea mays RepID=B6U2F0_MAIZE	-3.696
Ta.22954.1.S1_a_at	CA673824	Putative thionin Osthi1 n=2 Tax=Oryza sativa RepID=Q5Z4W6_ORYSJ	-3.839
Ta.28186.2.A1_a_at	CA671332	Voltage-dependent outwardly rectifying plasma membrane K+ channel KCO1/TPK1 n=4	-5.131
Ta.20696.3.S1_s_at	CA598744	Circumsporozoite protein n=2 Tax=Andropogoneae RepID=B6TF33_MAIZE	-6.406
TaAffx.36593.1.S1_at	BQ166396	Amylase inhibitor-like protein n=3 Tax=Triticum RepID=A9UID9_WHEAT	-8.802
TaAffx.116496.1.S1_at	CA693971	Phytochrome C n=10 Tax=Triticeae RepID=Q2I714_HORVD	-14.526
Ta.21379.1.S1_s_at	CA674527	Voltage-dependent outwardly rectifying plasma membrane K+ channel KCO1/TPK1 n=4	-15.607
Ta.28005.1.A1_at	CD862101	Phytochrome C (Fragment) n=1 Tax=Hordeum vulgare RepID=Q945T7_HORVU	-16.267
Ta.29481.1.S1_at	CK194207	Circumsporozoite protein n=2 Tax=Andropogoneae RepID=B6TF33_MAIZE	-169.064

Legend: The annotation is made according to Affymetrix Gene Chip® wheat genome array of the 94 miscellaneous probesetIDs differentially regulated complemented with BLAST results showing the Genbank accession number, UniProt or NCBI description and is presented in decreasing order of differential expression of $\geq$ 2-fold and $\leq$ -2-fold cut off.

Table S6:*mvp* wheat plant unknown genes differentially regulated identified by microarray

Affymetrix probeset IDs	GenBank Accession	UniProt and NCBI Description	Fold Change
Ta.8447.1.S1_a_at	CA669038	Putative uncharacterized protein n=1 Tax=Oryza sativa Indica Group RepID=B8AKC8_ORYSI	12.977
Ta.19723.1.S1_at	CA657361	wlm0.pk0034.h7 wlm0 Triticum aestivum cDNA clone wlm0.pk0034.h7 5' end	6.056
TaAffx.109794.1.S1_s_at	CA668708	Putative uncharacterized protein n=1 Tax=Oryza sativa Indica Group RepID=B8AKC8_ORYSI	5.763
Ta.62.1.S1_x_at	BM136002	Os01g0382000 protein n=5 Tax=Oryza sativa RepID=Q7F2P0_ORYSJ	5.324
TaAffx.110724.1.S1_at	CA655297	Putative uncharacterized protein n=1 Tax=Zea mays RepID=B8A3M2_MAIZE	5.219
Ta.28233.1.S1_at	CA599187	Putative uncharacterized protein Sb10g024350 n=1 Tax=Sorghum bicolor RepID=C5Z653_SORBI	5.214
Ta.23271.1.S1_s_at	CA680274	wlm24.pk0004.a5 wlm24 Triticum aestivum cDNA clone wlm24.pk0004.a5 5' end	5.156
Ta.8356.1.S1_at	BQ161783	Putative uncharacterized protein n=1 Tax=Oryza sativa Indica Group RepID=A2WKT6_ORYSI	5.002
Ta.30765.1.S1_at	CN011347	Putative uncharacterized protein Sb01g037040 n=1 Tax=Sorghum bicolor RepID=C5X1U8_SORBI	4.935
TaAffx.53867.1.S1_at	CA688277	wlm96.pk039.j13 wlm96 Triticum aestivum cDNA clone wlm96.pk039.j13 5' end	4.499
Ta.23271.2.S1_a_at	CA677139	wlm12.pk0009.c12 wlm12 Triticum aestivum cDNA clone wlm12.pk0009.c12 5' end	4.484
TaAffx.61466.1.S1_at	BJ251396	Putative uncharacterized protein Sb03g037575 n=1 Tax=Sorghum bicolor RepID=C5XN35_SORBI	4.472
TaAffx.97767.1.A1_at	CA721990	Putative uncharacterized protein Sb01g033530 n=1 Tax=Sorghum bicolor RepID=C5WXP8_SORBI	4.222
Ta.21340.1.S1_a_at	CA683606	Putative uncharacterized protein n=1 Tax=Oryza sativa Japonica Group RepID=Q6ZKP5_ORYSJ	4.203
Ta.8356.1.S1_s_at	BQ161783	Putative uncharacterized protein n=1 Tax=Oryza sativa Indica Group RepID=A2WKT6_ORYSI	4.191
TaAffx.7302.1.S1_at	CA662601	Os01g0678000 protein n=1 Tax=Oryza sativa Japonica Group RepID=Q5QM71_ORYSJ	4.055
TaAffx.27177.1.S1_at	CA680302	Putative uncharacterized protein Sb09g004960 n=1 Tax=Sorghum bicolor RepID=C5Z115_SORBI	3.985
Ta.23165.2.S1_x_at	CA667728	Triticum aestivum clone wlsu2.pk0001.h3:fis, full insert mRNA sequence	3.958
TaAffx.111759.3.S1_s_at	CA660613	wlm1.pk0023.g12 wlm1 Triticum aestivum cDNA clone wlm1.pk0023.g12 5' end	3.918
Ta.23165.3.S1_x_at	CA669496	Triticum aestivum clone wlsu2.pk0001.h3:fis, full insert mRNA sequence	3.912
TaAffx.43393.1.S1_at	BQ482808	Os02g0102900 protein n=4 Tax=Poaceae RepID=Q6ZFJ9_ORYSJ	3.861
Ta.192.1.S1_at	U32431	Putative uncharacterized protein n=1 Tax=Triticum aestivum RepID=Q41523_WHEAT	3.853
Ta.14779.1.S1_at	CA681945	Putative uncharacterized protein Sb01g027360 n=1 Tax=Sorghum bicolor RepID=C5WPZ1_SORBI	3.794
Ta.27503.1.A1_at	CA659276	Putative uncharacterized protein n=1 Tax=Oryza sativa Indica Group RepID=B8B370_ORYSI	3.794
Ta.12198.1.A1_at	BQ171803	WHE1659-1662_P05_P05ZT Wheat heat stressed flag leaf cDNA library Triticum aestivum	3.723
Ta.9430.1.S1_at	AJ614438	Putative uncharacterized protein Sb10g004170 n=1 Tax=Sorghum bicolor RepID=C5Z4H2_SORBI	3.685
Ta.19805.2.S1_a_at	CA683372	Os04g0168400 protein n=3 Tax=Oryza sativa RepID=B7F8V8_ORYSJ	3.659
Ta.12118.1.S1_a_at	CA698971	Putative uncharacterized protein n=1 Tax=Zea mays RepID=C0PFC3_MAIZE	3.606
TaAffx.97737.1.A1_at	CA722456	Triticum aestivum cDNA, clone: WT006_G18, cultivar: Chinese Spring	3.559
TaAffx.122333.1.S1_at	CA725295	Putative uncharacterized protein Sb03g045090 n=1 Tax=Sorghum bicolor RepID=C5XH43_SORBI	3.539
TaAffx.26668.1.S1_at	CA686407	Putative uncharacterized protein Sb01g027360 n=1 Tax=Sorghum bicolor RepID=C5WPZ1_SORBI	3.528
TaAffx.13303.1.S1_at	BG909514	Putative uncharacterized protein n=1 Tax=Oryza sativa Indica Group RepID=A2WKZ0_ORYSI	3.437
Ta.520.1.S1_at	BE604553	WHE1413-1416_P07_P07ZS Wheat drought stressed leaf cDNA library Triticum aestivum	3.429
TaAffx.108556.1.S1_at	CA692789	Putative uncharacterized protein n=2 Tax=Zea mays RepID=B4FS23_MAIZE	3.396
Ta.5766.1.S1_at	BJ289079	Os03g0218400 protein n=5 Tax=Poaceae RepID=Q10PW9_ORYSJ	3.376
Ta.7711.1.A1_at	BQ161212	Putative uncharacterized protein Sb08g017560 n=1 Tax=Sorghum bicolor RepID=C5YPJ9_SORBI	3.359
Ta.231.1.S1_x_at	AF079526	PR17c n=2 Tax=Triticeae RepID=A7YA60_HORVD	3.356
TaAffx.26815.1.S1_at	CA684496	BLN1-2 n=3 Tax=Hordeum vulgare subsp. vulgare RepID=B8X453_HORVD	3.336
TaAffx.128798.2.S1_x_at	CA695322	Putative uncharacterized protein Sb08g017540 n=1 Tax=Sorghum bicolor RepID=C5YPJ7_SORBI	3.319
Ta.3869.1.S1_at	CA674403	Os08g0127100 protein n=6 Tax=Poaceae RepID=Q6ZK52_ORYSJ	3.266

TaAffx.55188.1.S1_at	CA670456	wlsu1.pk026.n9 wlsu1 Triticum aestivum cDNA clone wlsu1.pk026.n9 5' end	3.249
Ta.6051.1.S1_a_at	CD914245	Os10g0416500 protein n=3 Tax=Oryza sativa RepID=Q9FYR9_ORYSJ	3.239
Ta.20549.1.S1_x_at	CA668159	Putative uncharacterized protein Sb04g030310 n=1 Tax=Sorghum bicolor RepID=C5Y093_SORBI	3.227
TaAffx.81496.1.S1_at	CA685342	Putative uncharacterized protein Sb05g019490 n=1 Tax=Sorghum bicolor RepID=C5Y384_SORBI	3.220
Ta.30860.1.S1_at	CN010964	WHE3878_C08_F16ZS Wheat Fusarium graminearum infected spike cDNA Triticum aestivum	3.203
Ta.27279.1.S1_at	BT009316	Os06g0330400 protein n=2 Tax=Oryza sativa RepID=Q69UX2_ORYSJ	3.114
TaAffx.108556.1.S1_x_at	CA692789	Putative uncharacterized protein n=2 Tax=Zea mays RepID=B4FS23_MAIZE	3.113
TaAffx.84552.1.S1_at	CA634441	wle1n.pk0086.g12 wle1n Triticum aestivum cDNA clone wle1n.pk0086.g12 5' end	3.096
TaAffx.71225.1.A1_at	BE492103	WHE0551_F06_F06ZE Triticum monococcum vegetative apex cDNA Triticum monococcum	3.073
Ta.21326.1.S1_a_at	AJ613350	Os07g0518100 protein n=3 Tax=Oryza sativa RepID=Q7EZ52_ORYSJ	3.052
Ta.10617.1.S1_at	BQ166125	Os01g0108400 protein n=4 Tax=Oryza sativa RepID=Q0JRC6_ORYSJ	3.002
Ta.6051.3.S1_x_at	CA717933	Os10g0416500 protein n=3 Tax=Oryza sativa RepID=Q9FYR9_ORYSJ	2.956
Ta.28233.2.S1_a_at	CA695499	Putative uncharacterized protein Sb10g024350 n=1 Tax=Sorghum bicolor RepID=C5Z653_SORBI	2.934
TaAffx.114390.1.S1_at	CA593923	Putative uncharacterized protein n=2 Tax=Zea mays RepID=B6SJC9_MAIZE	2.750
Ta.13991.1.S1_x_at	CA666889	Putative uncharacterized protein Sb03g043610 n=1 Tax=Sorghum bicolor RepID=C5XG61_SORBI	2.750
Ta.9332.1.S1_x_at	BQ789066	Os09g0441400 protein n=2 Tax=Oryza sativa RepID=Q69P73_ORYSJ	2.643
Ta.3869.2.S1_at	CA735969	Triticum aestivum cDNA, clone: WT008_I09, cultivar: Chinese Spring	2.636
Ta.20549.1.S1_s_at	CA668159	Putative uncharacterized protein Sb04g030310 n=1 Tax=Sorghum bicolor RepID=C5Y093_SORBI	2.621
Ta.12434.1.S1_at	CD862952	OSJNBa0086B14.7 protein n=1 Tax=Oryza sativa Japonica Group RepID=Q7XV48_ORYSJ	2.618
TaAffx.81921.1.S1_at	CA680100	Os01g0613500 protein n=2 Tax=Oryza sativa RepID=Q9FTI3_ORYSJ	2.617
TaAffx.15958.1.S1_at	BQ801634	Os01g0914100 protein n=2 Tax=Oryza sativa RepID=Q8S077_ORYSJ	2.595
Ta.13956.1.S1_at	BJ287532	cDNA library, Wh_r Triticum aestivum cDNA clone whr20i23 3'	2.559
Ta.10581.1.A1_at	CK156211	Hordeum vulgare subsp. vulgare cDNA clone: FLbaf14j06, mRNA sequence	2.552
TaAffx.24109.1.A1_at	CK216113	Os03g0400200 protein n=4 Tax=Oryza sativa RepID=Q10K20_ORYSJ	2.547
Ta.231.1.S1_at	AF079526	PR17c n=2 Tax=Triticeae RepID=A7YA60_HORVD	2.547
TaAffx.124475.1.A1_at	CK212125	BLT14.2 protein n=2 Tax=Hordeum vulgare RepID=Q40033_HORVU	2.528
Ta.11397.1.A1_at	BQ170011	Putative uncharacterized protein Sb02g035400 n=1 Tax=Sorghum bicolor RepID=C5XAR6_SORBI	2.498
Ta.30913.1.A1_at	CN012655	Os09g0454600 protein n=3 Tax=Oryza sativa RepID=Q0J198_ORYSJ	2.461
Ta.22628.1.S1_x_at	CA690208	Putative uncharacterized protein n=1 Tax=Zea mays RepID=B6SP49_MAIZE	2.453
Ta.22628.1.S1_at	CA690208	Putative uncharacterized protein n=1 Tax=Zea mays RepID=B6SP49_MAIZE	2.436
TaAffx.109085.1.S1_at	CA680382	wlm24.pk0006.g11 wlm24 Triticum aestivum cDNA clone wlm24.pk0006.g11 5' end	2.417
TaAffx.99316.1.A1_at	CK207671	Putative uncharacterized protein n=1 Tax=Oryza sativa Japonica Group RepID=B9GE50_ORYSJ	2.412
Ta.20197.1.S1_at	CA674805	Putative uncharacterized protein (Fragment) n=2 Tax=Papilionoideae RepID=B7FFL2_MEDTR	2.410
TaAffx.34169.1.S1_at	BJ287371	Putative uncharacterized protein n=2 Tax=Oryza sativa RepID=B9FBJ1_ORYSJ	2.410
Ta.11421.1.A1_at	BQ170075	Os01g0266500 protein n=1 Tax=Oryza sativa Japonica Group RepID=Q9SDD7_ORYSJ	2.401
Ta.25487.1.S1_at	CD373987	Putative uncharacterized protein Sb02g035450 n=1 Tax=Sorghum bicolor RepID=C5XAS1_SORBI	2.385
Ta.25539.1.S1_at	CA678031	BLT14.1 protein n=2 Tax=Triticeae RepID=Q40032_HORVU	2.362
Ta.4696.1.S1_at	BJ275186	Os01g0366300 protein n=1 Tax=Oryza sativa Japonica Group RepID=Q5Z8B7_ORYSJ	2.353
TaAffx.51261.1.S1_at	CA728497	Os01g0793900 protein n=2 Tax=Oryza sativa RepID=Q8S1K8_ORYSJ	2.329
Ta.19786.1.A1_at	CA658969	Putative uncharacterized protein Sb01g016640 n=1 Tax=Sorghum bicolor RepID=C5WV89_SORBI	2.294
Ta.25754.1.A1_at	CD373766	Putative uncharacterized protein Sb03g045850 n=1 Tax=Sorghum bicolor RepID=C5XHV9_SORBI	2.291
Ta.3162.1.S1_at	BJ215513	Putative uncharacterized protein n=1 Tax=Oryza sativa Japonica Group RepID=B9FSW5_ORYSJ	2.286
Ta.25542.1.S1_at	CD896341	Os02g0712700 protein n=3 Tax=Oryza sativa RepID=Q0DY65_ORYSJ	2.273

Ta.8399.2.S1_at	CA694080	Putative uncharacterized protein Sb03g028700 n=1 Tax=Sorghum bicolor RepID=C5XEF4_SORBI	2.266
Ta.8512.1.S1_at	BQ162001	Os07g0631700 protein n=2 Tax=Oryza sativa RepID=Q8LHN5_ORYSJ	2.254
TaAffx.82108.1.S1_x_at	CA677464	Os01g0115700 n=1 Tax=Oryza sativa Japonica Group RepID=UPI0000DD891C	2.249
Ta.24254.2.S1_at	BQ800827	Os03g0290300 protein (Fragment) n=1 Tax=Oryza sativa Japonica Group RepID=Q0DSS9_ORYSJ	2.246
Ta.13232.2.S1_at	BQ166297	WHE0840_F03_L06ZT Wheat vernalized crown cDNA library Triticum aestivum	2.240
TaAffx.131248.2.S1_at	CA670789	Hordeum vulgare subsp. vulgare cDNA clone: FLbaf2f01, mRNA sequence	2.223
TaAffx.83027.1.S1_at	CA662924	Os05g0135100 protein n=1 Tax=Oryza sativa Japonica Group RepID=Q0DKY8_ORYSJ	2.221
Ta.4921.1.S1_at	BE438217	Putative uncharacterized protein n=1 Tax=Oryza sativa Japonica Group RepID=B9FAG9_ORYSJ	2.216
Ta.15129.1.S1_at	CA690804	wlm96.pk052.p8 wlm96 Triticum aestivum cDNA clone wlm96.pk052.p8 5' end	2.215
TaAffx.28302.4.S1_at	CA662104	wlmk1.pk0015.d5 wlmk1 Triticum aestivum cDNA clone wlmk1.pk0015.d5 5' end	2.208
Ta.24832.1.S1_s_at	CA668285	Os08g0127100 protein n=6 Tax=Poaceae RepID=Q6ZK52_ORYSJ	2.182
TaAffx.16900.1.A1_at	CK215179	Putative uncharacterized protein n=1 Tax=Oryza sativa Japonica Group RepID=Q7X669_ORYSJ	2.180
Ta.22625.1.A1_s_at	CA666706	Os02g0634700 protein n=2 Tax=Oryza sativa RepID=Q6H7I7_ORYSJ	2.179
Ta.21035.1.S1_at	CA614540	Putative uncharacterized protein Sb03g006590 n=1 Tax=Sorghum bicolor RepID=C5XQN8_SORBI	2.166
TaAffx.131248.2.S1_s_at	CA670789	Hordeum vulgare subsp. vulgare cDNA clone: FLbaf2f01, mRNA sequence	2.165
TaAffx.21249.1.S1_at	CA706624	Os02g0720600 protein n=2 Tax=Oryza sativa RepID=Q6Z674_ORYSJ	2.162
Ta.12671.1.S1_a_at	CK194385	Putative uncharacterized protein Sb04g024020 n=1 Tax=Sorghum bicolor RepID=C5XVR6_SORBI	2.160
TaAffx.5899.1.S1_at	CA696250	Putative uncharacterized protein n=1 Tax=Oryza sativa Indica Group RepID=A2YM74_ORYSI	2.156
TaAffx.108908.1.S1_x_at	CA684616	Os03g0663500 protein (Fragment) n=3 Tax=Oryza sativa RepID=Q0DPU1_ORYSJ	2.153
Ta.27455.1.S1_at	CA644954	Putative uncharacterized protein Sb01g027360 n=1 Tax=Sorghum bicolor RepID=C5WPZ1_SORBI	2.152
Ta.12441.1.A1_at	BQ172342	Putative uncharacterized protein n=3 Tax=Zea mays RepID=C4JAI5_MAIZE	2.137
Ta.8076.1.S1_at	CA684491	Os02g0634700 protein n=2 Tax=Oryza sativa RepID=Q6H7I7_ORYSJ	2.135
Ta.28224.1.S1_x_at	CK193135	Os01g0384800 protein n=2 Tax=Oryza sativa RepID=Q5VNY3_ORYSJ	2.134
TaAffx.21249.1.S1_x_at	CA706624	Os02g0720600 protein n=2 Tax=Oryza sativa RepID=Q6Z674_ORYSJ	2.133
TaAffx.82108.1.S1_at	CA677464	Os01g0115700 n=1 Tax=Oryza sativa Japonica Group RepID=UPI0000DD891C	2.131
Ta.5824.2.S1_x_at	AL830800	Os02g0740600 protein n=2 Tax=Oryza sativa RepID=Q6Z7S9_ORYSJ	2.124
TaAffx.54530.1.S1_at	CA633759	Os04g0103500 protein n=2 Tax=Oryza sativa RepID=Q7XMR2_ORYSJ	2.122
Ta.8323.1.A1_at	BQ161741	Os02g0515200 protein n=2 Tax=Oryza sativa RepID=Q67UP1_ORYSJ	2.090
TaAffx.83591.1.S1_at	CA655732	Putative uncharacterized protein n=1 Tax=Oryza sativa Indica Group RepID=B8B433_ORYSI	2.087
TaAffx.8335.1.S1_at	CA627027	wl1n.pk151.c8 wl1n Triticum aestivum cDNA clone wl1n.pk151.c8 5' end	2.068
Ta.25845.1.S1_at	CD453621	Putative uncharacterized protein Sb09g003460 n=1 Tax=Sorghum bicolor RepID=C5Z001_SORBI	2.052
TaAffx.15674.1.A1_at	CD490328	WHE2494_H02_P04ZT Triticum monococcum DV92 early reproductive apex	2.044
Ta.4831.1.S1_at	AJ611741	Putative uncharacterized protein Sb06g032110 n=1 Tax=Sorghum bicolor RepID=C5YA11_SORBI	2.042
Ta.1830.2.S1_x_at	CA683961	Putative uncharacterized protein Sb01g030980 n=1 Tax=Sorghum bicolor RepID=C5WUJ5_SORBI	2.037
Ta.21711.1.S1_at	CD883645	Putative uncharacterized protein Sb07g020050 n=1 Tax=Sorghum bicolor RepID=C5YKT1_SORBI	2.028
TaAffx.31754.1.S1_at	CA608501	Putative uncharacterized protein n=1 Tax=Oryza sativa Japonica Group RepID=B9FXP2_ORYSJ	2.020
TaAffx.70641.1.S1_at	BQ802487	WHE2826_E02_I04ZS Triticum monococcum vernalized apex	2.018
TaAffx.4290.1.A1_at	CA681239	wlm24.pk0014.c7 wlm24 Triticum aestivum cDNA clone wlm24.pk0014.c7 5' end.	2.001
TaAffx.130076.1.S1_at	BQ168759	Os05g0550600 protein n=2 Tax=Oryza sativa RepID=Q6L4G9_ORYSJ	-2.002
TaAffx.18447.3.S1_s_at	CA737476	Putative uncharacterized protein Sb08g020600 n=1 Tax=Sorghum bicolor	-2.022
Ta.2926.1.A1_at	BJ207389	Triticum aestivum cDNA, clone: WT009_B01, cultivar: Chinese Spring	-2.022
Ta.28728.1.S1_at	AF139815	Aquaporin PIP2-4 n=9 Tax=Poaceae RepID=PIP24_MAIZE	-2.035
Ta.8640.1.S1_a_at	BQ807183	Os07g0169600 protein n=2 Tax=Oryza sativa RepID=Q69LD9_ORYSJ	-2.037

Ta.6558.1.S1_x_at	CK208447	Os03g0787200 protein n=2 Tax=Oryza sativa RepID=Q6F3B1_ORYSJ	-2.042
Ta.28847.1.S1_a_at	CA740446	Os01g0795100 protein n=2 Tax=Oryza sativa Japonica Group RepID=Q8S1I0_ORYSJ	-2.056
Ta.20938.2.A1_x_at	CA624118	Os01g0117900 protein n=2 Tax=Oryza sativa RepID=Q9FTZ6_ORYSJ	-2.066
TaAffx.63920.1.A1_x_at	BQ162587	Hordeum vulgare subsp. vulgare cDNA clone: FLbaf149n21, mRNA sequence	-2.084
Ta.3857.1.A1_at	CA698230	Os08g0137400 protein n=3 Tax=Oryza sativa RepID=Q0J845_ORYSJ	-2.090
TaAffx.8097.1.S1_x_at	CA636056	wle1n.pk0106.b2 wle1n Triticum aestivum cDNA clone wle1n.pk0106.b2 5' end	-2.125
Ta.28422.1.A1_s_at	BJ317142	Os03g0724600 protein (Fragment) n=3 Tax=Oryza sativa RepID=Q0DP01_ORYSJ	-2.141
Ta.7724.3.S1_at	CA631315	Putative uncharacterized protein Sb09g001060 n=1 Tax=Sorghum bicolor	-2.145
Ta.8710.1.A1_at	BQ162259	Triticum aestivum cDNA, clone: WT012_L15, cultivar: Chinese Spring	-2.167
Ta.6934.1.A1_x_at	CK209084	UPI0000D8C4F1 related cluster n=1 Tax=Danio rerio RepID=UPI0000D8C4F1	-2.170
Ta.20540.3.S1_x_at	BJ318550	Putative uncharacterized protein Sb01g004270 n=2 Tax=Andropogoneae	-2.210
Ta.20540.3.S1_at	BJ318550	Putative uncharacterized protein Sb01g004270 n=2 Tax=Andropogoneae	-2.229
Ta.11684.1.A1_at	BQ170589	Os03g0234900 protein n=2 Tax=Oryza sativa RepID=Q5U1Q4_ORYSJ	-2.240
Ta.20938.1.A1_at	BQ169082	Os01g0117900 protein n=2 Tax=Oryza sativa RepID=Q9FTZ6_ORYSJ	-2.241
Ta.20938.1.A1_a_at	BQ169082	Os01g0117900 protein n=2 Tax=Oryza sativa RepID=Q9FTZ6_ORYSJ	-2.271
TaAffx.16068.1.S1_at	BQ168935	Oryza sativa Japonica Group cDNA clone:002-112-B01, full insert sequence	-2.273
Ta.7724.3.S1_x_at	CA631315	Putative uncharacterized protein Sb09g001060 n=1 Tax=Sorghum bicolor	-2.281
Ta.14454.1.S1_s_at	BJ215196	Os09g0433800 protein n=3 Tax=Oryza sativa RepID=Q69PH9_ORYSJ	-2.293
Ta.5563.1.S1_at	CA643873	Putative uncharacterized protein OSJNBa0027N19.12 n=2 Tax=Oryza sativa	-2.341
Ta.6934.1.A1_a_at	CK209084	UPI0000D8C4F1 related cluster n=1 Tax=Danio rerio RepID=UPI0000D8C4F1	-2.385
Ta.2636.1.S1_x_at	CA605844	Putative uncharacterized protein Sb09g024850 n=2 Tax=Andropogoneae	-2.397
Ta.28847.1.S1_at	CA740446	Os01g0795100 protein n=2 Tax=Oryza sativa Japonica Group RepID=Q8S1I0_ORYSJ	-2.416
Ta.26213.1.S1_at	CD452838	Putative uncharacterized protein Sb06g022610 n=2 Tax=Andropogoneae	-2.418
Ta.23219.1.A1_x_at	CA737258	wpi2s.pk001.n10 wpi2s Triticum aestivum cDNA clone wpi2s.pk001.n10 5' end	-2.422
Ta.22954.3.S1_at	CA632583	Triticum aestivum cultivar Renan clone BAC 930H14, complete sequence	-2.441
TaAffx.84069.1.S1_at	CA645540	Os03g0859100 protein n=2 Tax=Oryza sativa RepID=Q84M86_ORYSJ	-2.501
Ta.24294.1.A1_at	BQ172428	Triticum aestivum cDNA clone WHE2064_H07_O14.	-2.693
Ta.1037.1.S1_at	CA638909	Os01g0731100 protein n=4 Tax=Oryza sativa RepID=Q94EA4_ORYSJ	-2.700
TaAffx.16307.1.S1_at	BQ800749	Hordeum vulgare subsp. vulgare cDNA clone: FLbaf143f08, mRNA sequence	-2.716
Ta.3304.1.S1_at	BJ243222	Putative uncharacterized protein Sb05g001550 n=1 Tax=Sorghum bicolor	-2.804
Ta.4455.1.A1_at	BJ253690	Os01g0842500 protein n=2 Tax=Oryza sativa RepID=Q8S2A8_ORYSJ	-2.810
Ta.6965.1.S1_at	BJ313225	Wh_yf Triticum aestivum cDNA clone whyf4e06 5'.	-2.845
Ta.3813.1.A1_at	BQ238416	Putative uncharacterized protein Sb05g026610 n=1 Tax=Sorghum bicolor	-3.158
Ta.29640.1.S1_x_at	CK205489	Putative uncharacterized protein n=2 Tax=Zea mays RepID=B7ZYD6_MAIZE	-3.213
Ta.10151.1.S1_at	BJ234600	Putative uncharacterized protein Sb09g016470 n=2 Tax=Sorghum bicolor	-3.503
Ta.556.1.S1_x_at	CA671780	Os05g0153300 protein n=3 Tax=Oryza sativa RepID=Q65XP3_ORYSJ	-3.531
Ta.556.1.S1_at	CA671780	Os05g0153300 protein n=3 Tax=Oryza sativa RepID=Q65XP3_ORYSJ	-3.849
Ta.28480.1.S1_s_at	CD452788	Os03g0111200 protein n=3 Tax=Oryza sativa RepID=Q10SU0_ORYSJ	-4.001
Ta.11506.1.S1_a_at	CK215703	Os07g0617500 protein n=2 Tax=Oryza sativa RepID=Q8GS08_ORYSJ	-4.843
TaAffx.85922.1.S1_x_at	CA618396	Putative uncharacterized protein (Fragment) n=5 Tax=Triticeae RepID=Q9M540_AGRCR	-11.074
Ta.7832.1.S1_at	CA646083	Putative uncharacterized protein Sb01g007930 n=2 Tax=Poaceae RepID=C5X0B2_SORBI	-114.258
TaAffx.85922.1.S1_s_at	CA618396	Putative uncharacterized protein Sb01g007930 n=2 Tax=Poaceae RepID=C5X0B2_SORBI	-114.866

Legend: The annotation is made according to Affymetrix Gene Chip® wheat genome array of the 168 Unknown probesetIDs differentially complemented with BLAST results showing the Genbank accession number, UniProt or NCBI description and is presented in decreasing order of differential expression of ≥ 2-fold and $\leq$ -2-fold cut off.

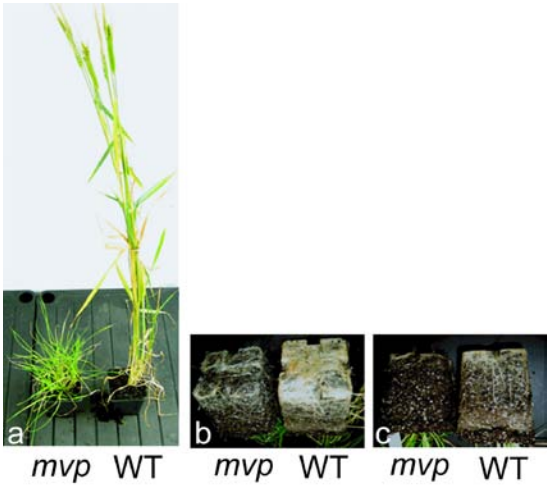

Figure S1

Figure S1: Phenotype of maintained vegetative phase (*mvp*) plants and control plants (WT). Pictures were taken after 3 months of growth at 20°C under LD conditions to show the difference of development between *mvp*mutant (*mvp*) and wild typeplants (WT). a: whole plant;b: root architecture (bottom face); c: root architecture(side face).

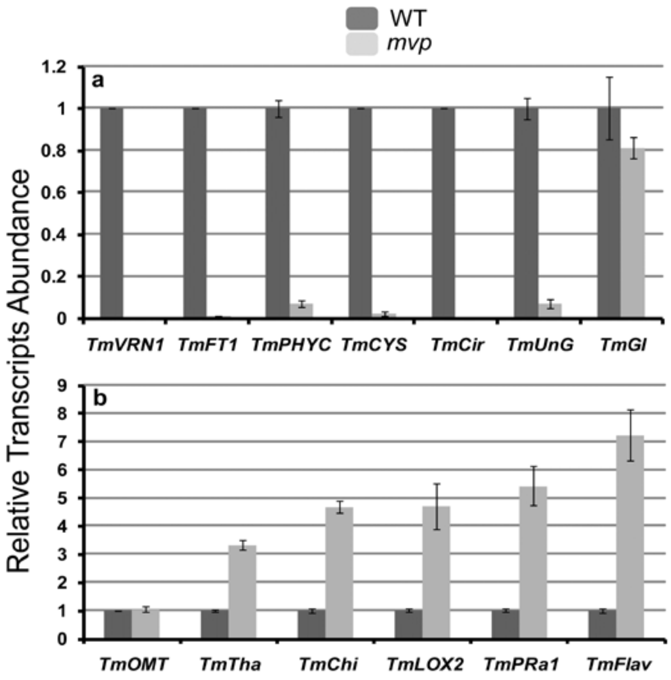

Figure S2

Figure S2: Validation of microarray results with selected genes using qRT-PCR experiment in *mvp* and wild type control plants

a) down-regulated and control genes; **b**) up-regulated and control genes. Relative transcript abundance was calculated and normalized with respect to 18S *TaRNA* for the qRT-PCR experiment. Data represent the mean ± SEM from three biological replicates. Each replicate (R1, R2 and R3) was obtained from five mutants plants (*mvp*) and from three control wild type plants. The RNA samples are the same used in Figure 1 and the microarray experiment. In addition, we repeated the same experiment with three new biological replicates with similar results.

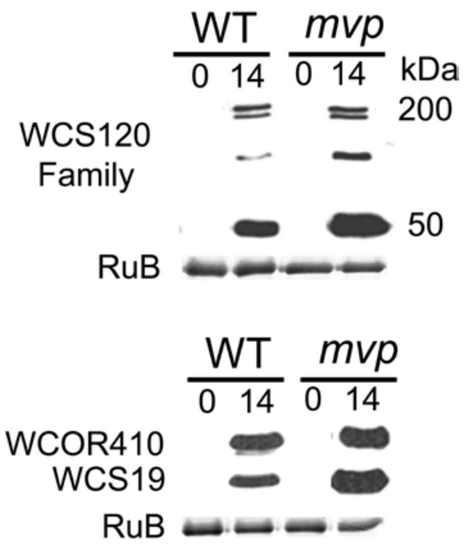

Figure S3

Figure S3: Expression level of COR proteins in wild-type and *mvp* mutant plants before and after cold acclimation analysed by western blot.After 3 weeks of germination at 20°C under LD conditions (0), Wild-type eikorn wheat (WT) and *mvp* mutant (*mvp*) plants were cold-acclimated for 14 days (14) under LD conditionsat 4°C. Equal amounts of total soluble proteins from whole plant tissues were analysed. Proteins were separated on a 12% SDS–PAGE and transferred to a polyvinylidene difluoride membrane, and an anti-WCS120, anti-WCOR410, anti-WCS19 antibodies were used to detect the corresponding proteins. Rubisco (RuB) was used as load control.

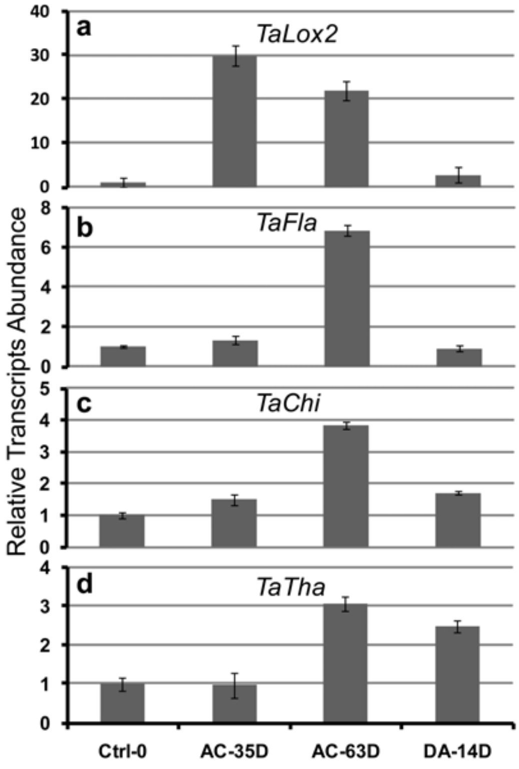

Figure S4

Figure S4: Relative expression level of *TaLox2*, *TaFla*, *TaChi* and *TaTha* during vernalization and deacclimation conditions in hexaploid wheat seedlings analyzed by qRT-PCR.After 2 weeks of germination at 20°C under LD conditions, non-vernalized winter (cv Norstar) wheat plants were vernalized under SD conditions at 4°C for 63 days and deacclimated for 14 days at 20°C under LD conditions. The aerial part was sampled around 4 hours after the beginning of the daylight period. The expression level of *TaLox2* (panel **a**), *TaFla*(panel **b**), *TaChi* (panel **c**) and *TaTha* (panel **d**) are expressed relative to the non-vernalized point (Ctrl-0). Data represent the mean ± SEM from 4 biological replicates. The level is normalized using*TaRNA 18S*.

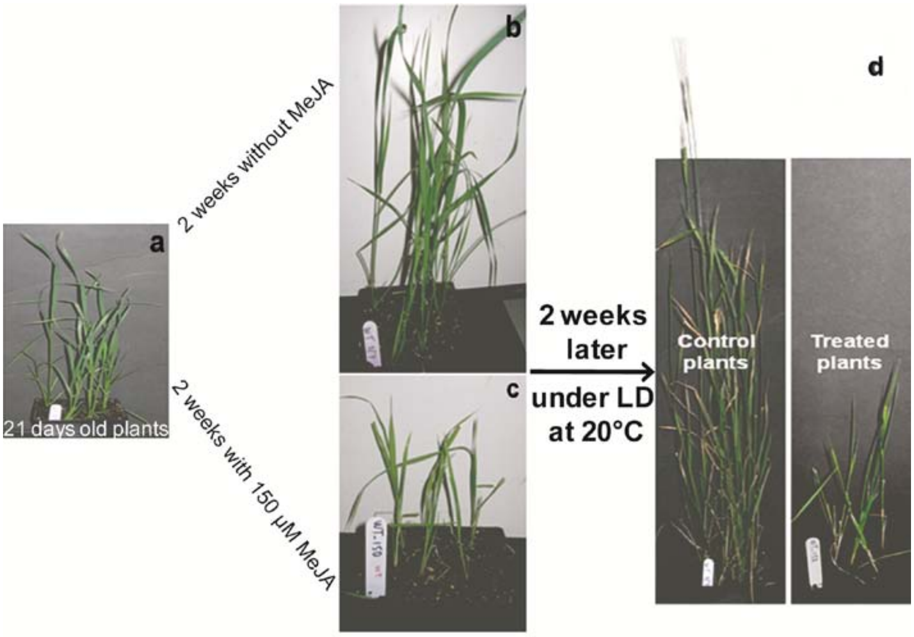

Figure S5

Figure S5: Effect of MeJA treatment on plant development in wild typeeinkornwheat. Plants phenotypes were analysed before and after MeJA treatment. a) wild type einkorn wheat plants before treatment (after three weeks of germination at 20°C under LD conditions; b) control plants (treated with 0,1%tween 20 solution only) were kept under LD conditions at 20°C for two weeks; c) treated plants were sprayed with 150 µM of MeJA dissolved in 0.1% tween 20 every day for two weeks under the same growth conditions and d) control and MeJA treated plants shown two weeks after the end of the treatment.

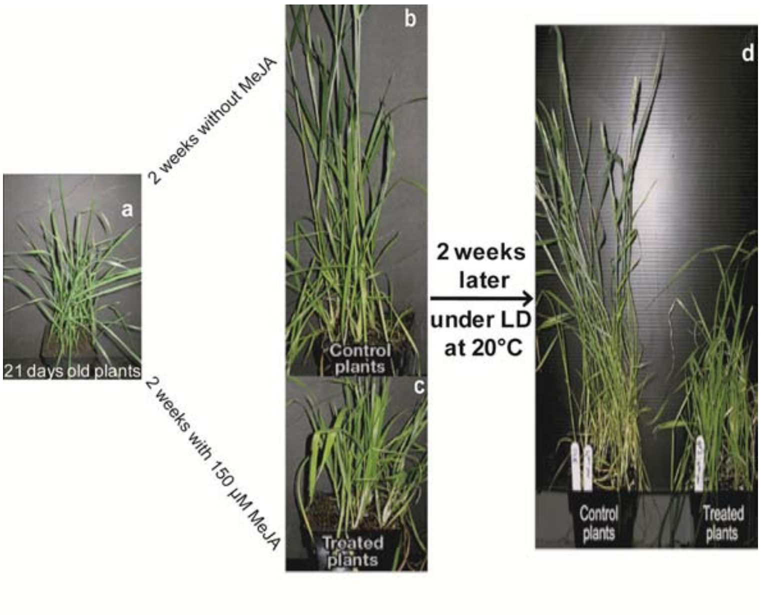

Figure S6

Figure S6: Effect of MeJA treatment on plant development in *Triticum aestivum* wheat cv Manitou. Plant phenotypes were analysed before and after MeJA treatment. **a)** spring wheat (cv Manitou) before treatment (after three weeks of germination at 20°C under LD conditions; **b**) control plants (treated with 0,1% tween 20 solution only) were kept under LD conditions at 20°C for two weeks; **c**) treated plants were sprayed with 150 µM of MeJA dissolved in 0.1% tween 20every day for two weeks under the same growth conditions and **d**) control and MeJA treated plants shown two weeks after the end oftreatment.

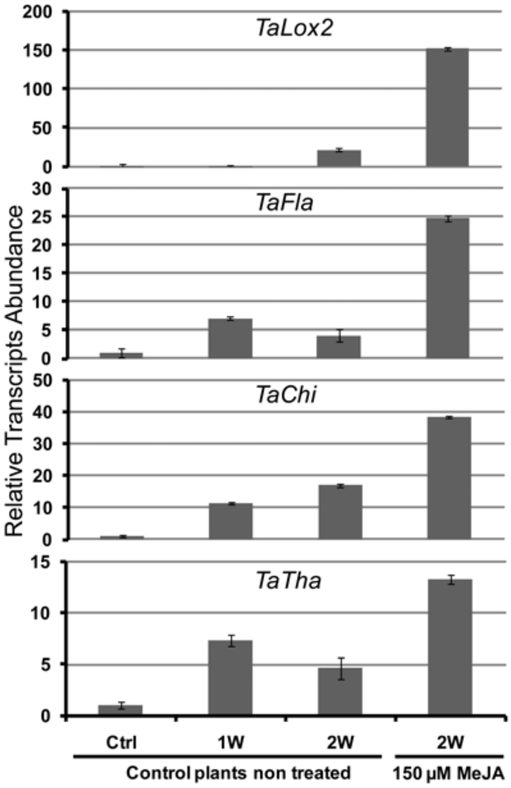

Figure S7

Figure S7: Effect of MeJA treatment on the expression of PR genes *TaLox2, TaFla, TaChi* and *TaTha* analyzed by qRT-PCR.The expression level of *TaLox2* (panel **a**), *TaFla*(panel **b**), *TaChi* (panel **c**) and *TaTha* (panel **d**) are expressed relative to the non-treated (Ctrl). Three weeks after germination at 20°C under LD conditions, control spring wheat (cv Manitou) plants (sprayed with 0.1% tween 20 solution only: Ctrl) were grown under LD conditions at 20°C for two weeks. Treated plants were sprayed with 150 µM of MeJA dissolved in 0.1% tween solution every day for 2 weeks under the same growth conditions. Total RNA was extracted from aerial parts and analyzed by qRT-PCR. The level is normalized by using *TaRNA18S*.Data represent the mean ± SEMfrom three biological replicates.
